# Immune factors produced by PBMCs upon stimulation with *lactobacillus delbrueckii* ssp. *bulgaricus* OLL1073R-1-derived exopolysaccharides inhibit HCoV-229E and SARS-CoV-2 replication

**DOI:** 10.1038/s41598-025-17308-3

**Published:** 2025-08-27

**Authors:** Shuyi Tang, Reiko Takai-Todaka, Shoko Ishii, Hiroki Kono, Reiko Watanabe, Miho Ogawa, Hiroshi Kano, Kazuhiko Katayama, Toshihiro Sashihara, Kenichi Hojo, Kei Haga

**Affiliations:** 1 Food Microbiology and Function Research Laboratories, R&D division, Meiji Co., Ltd., 1-29-1 Nanakuni, Hachioji, Tokyo, 192-0919 Japan; 2 Laboratory of Viral Infection Control, Ōmura Satoshi Memorial Institute, Graduate School of Infection Control Sciences, Kitasato University, 5-9-1, Shirokane, Minato-ku, Tokyo, 108-8641 Japan; 3 Wellness Science Labs, Meiji Holdings Co., Ltd., 1-29-1 Nanakuni, Hachioji, Tokyo, 192-0919 Japan; 4 Present Address: Health Science Research Unit, R&D Division, Meiji Co., Ltd., 1- 29-1 Nanakuni, Hachioji, Tokyo, 192-0919 Japan; 5 Present Address: Wellness Science Labs, Meiji Holdings Co., Ltd., 1-29-1 Nanakuni, Hachioji, Tokyo, 192-0919 Japan

**Keywords:** SARS-CoV-2, Human coronavirus, COVID-19, *Lactobacillus delbrueckii* ssp. *bulgaricus* OLL1073R-1, Exopolysaccharides, SARS-CoV-2, Antimicrobial responses

## Abstract

The COVID-19 pandemic entered a controllable phase due to vaccine and drug development. The development of defense strategies of enhancing host innate immunity remains imperative to counter the emergence of infectious SARS-CoV-2 variants. In this study, we examined the inhibitory effects of *Lactobacillus delbrueckii* ssp. *bulgaricus* OLL1073R-1 (OLL1073R-1)-derived exopolysaccharides (R-1 EPS), a natural immunomodulator, against coronaviral infection. The inhibitory effect of R-1 EPS on HCoV-229E was evaluated first and subsequently extended to SARS-CoV-2. Cell culture supernatant was obtained by stimulating human peripheral blood mononuclear cells with R-1 EPS (R-1 sup), and the inhibitory effect of R-1 sup on coronaviruses was evaluated using the human lung cell line, MRC5. High levels of proinflammatory cytokines were detected in R-1 sup, and replication of both HCoV-229E and SARS-CoV-2 was significantly suppressed in MRC5 cells pretreated with R-1 sup. Transcriptomic analysis revealed that type I IFN signaling was upregulated in R-1 sup-treated MRC5 cells. Our results suggest that immune factors, including proinflammatory cytokines, in R-1 sup, enhance type I IFN response, resulting in an inhibitory effect on both HCoV-229E and SARA-CoV-2. Although this study is an in vitro investigation and requires further in vivo validation, the findings provide a scientific basis for the application of OLL1073R-1 and R-1 EPS as preventive strategies against HCoV-229E and SARS-CoV-2.

**Supplementary Information:**

The online version contains supplementary material available at 10.1038/s41598-025-17308-3.

## Introduction

Since the initial reports of infection at the end of 2019, coronavirus disease 2019 (COVID-19), caused by the severe acute respiratory syndrome coronavirus 2 (SARS-CoV-2), has rapidly spread worldwide, leading to a significant number of infections, severe cases, and fatalities^1^. During this period, various treatment modalities and therapeutic drugs for COVID-19 have been developed, and vaccination efforts have progressed, with 67% of the global population receiving vaccinations at least once by December 2023^2^. As a result of the development of countermeasures against infectious diseases, the mortality rate associated with COVID-19 has dropped below 1%^2^. However, the viral properties of SARS-CoV-2, including infectivity and virulence, have not diminished. It continues to spread and propagate in human society, establishing itself as an enduring threat^3^. In such an environment, in addition to infection prevention and treatment through vaccines and therapeutic drugs, maintaining and enhancing individual-level defense against COVID-19 is crucial.

Seven infectious human coronaviruses have been identified. Among these, the severe acute respiratory syndrome coronavirus (SARS-CoV), Middle East respiratory syndrome (MERS)-CoV, and SARS-CoV-2 can lead to severe pneumonia. Human coronaviruses (HCoV)- 229E, NL63, OC43, and HKU1 often cause milder symptoms and are commonly associated with seasonal cold^4,5^; however, in rare cases, they may cause severe respiratory disease^6,7^. In case of SARS-CoV-2, the frequent emergence of more transmissible variants has complicated infection prevention efforts, including the need for ongoing booster vaccinations^8,9^. In this challenging context, it is important to develop defense strategies that exhibit broad-spectrum antiviral effects.

Food-derived natural immunomodulatory ingredients are attracting attention as means of activating the host’s innate immunity and developing antiviral defense. *Lactobacillus delbrueckii* ssp. *bulgaricus* OLL1073R-1 (OLL1073R-1) is used as a starter in yogurt and produces exopolysaccharides (R-1 EPS) with immunomodulatory effects^10,11^. Several clinical studies suggest a potential inhibitory effect of yogurt fermented with OLL1073R-1 against infections. The effects include, reducing the risk of catching the common cold^12^, enhancing dendritic cell^13^ and natural killer (NK) cell activities^12^, and augmenting antibody production in serum and saliva^14–16^. However, no prior studies have investigated the anti-coronavirus effects of OLL1073R-1. Regarding R-1 EPS, it enhances NK cells activity in vivo^17^ and exhibits antiviral effects against influenza viruses in both in vivo and in vitro settings^18,19^. Furthermore, R-1 EPS modulates the innate immune response triggered by the stimulation of Toll-like receptor (TLR) 3 and enhances type I interferon (IFN) production in epithelial cells in vitro^20^. In this study, we focused on R-1 EPS as an immunomodulator for activating innate immunity, which is expected to confer broad protection against various viral strains, including coronaviruses.

To develop preventive strategies against SARS-CoV-2 and other coronaviral infections, in this study, we initially investigated the inhibitory effect of R-1 EPS on HCoV-229E and subsequently extended our study to SARS-CoV-2. The mechanisms in which orally administered R-1 EPS modulates immune function in vivo remain unclear, but studies using mice have shown that oral administration of R-1 EPS enhances NK cell activity in the spleen^17,18^ and increases antibody production in the bronchi^18^. Additionally, clinical studies have demonstrated that consuming yogurt fermented with OLL1073R-1 leads to an increase in peripheral blood NK activity^12^ and dendritic cell activity^13^. Based on these studies, we hypothesized that oral administration of R-1 EPS exerts its immunomodulatory effect by initially interacting with immune cells in the intestinal mucosa or circulating blood and subsequently stimulating the immune response in peripheral tissues via cytokine production and immune cell migration. To examine the antiviral effects of R-1 EPS against coronaviruses, we established an in vitro experimental model using human peripheral blood mononuclear cells (PBMCs) and human lung cell line MRC5. Our results showed that immune factors derived from R-1 EPS-stimulated PBMCs effectively suppressed both HCoV-229E and SARS-CoV-2 replication in MRC5 cells. The mechanisms underlying this suppression were explored by transcriptomic analysis. This study provides a scientific basis for the use of R-1 EPS or yogurt fermented with OLL1073R-1, as a preventive strategy against infections by HCoV-229E, SARS-CoV-2, and other coronaviruses.

## Results

### R-1 EPS promotes proinflammatory cytokines and chemokines production in PBMCs

To examine the antiviral effect of R-1 EPS against coronavirus and explore the underlying mechanisms, an in vitro experimental model using human PBMCs and the human lung cell line MRC5 was established. First, PBMCs were stimulated with R-1 EPS to produce immune factors including cytokines and chemokines, and then, the antiviral effect of these immune factors against coronavirus were examined.

**Fig. 1:**
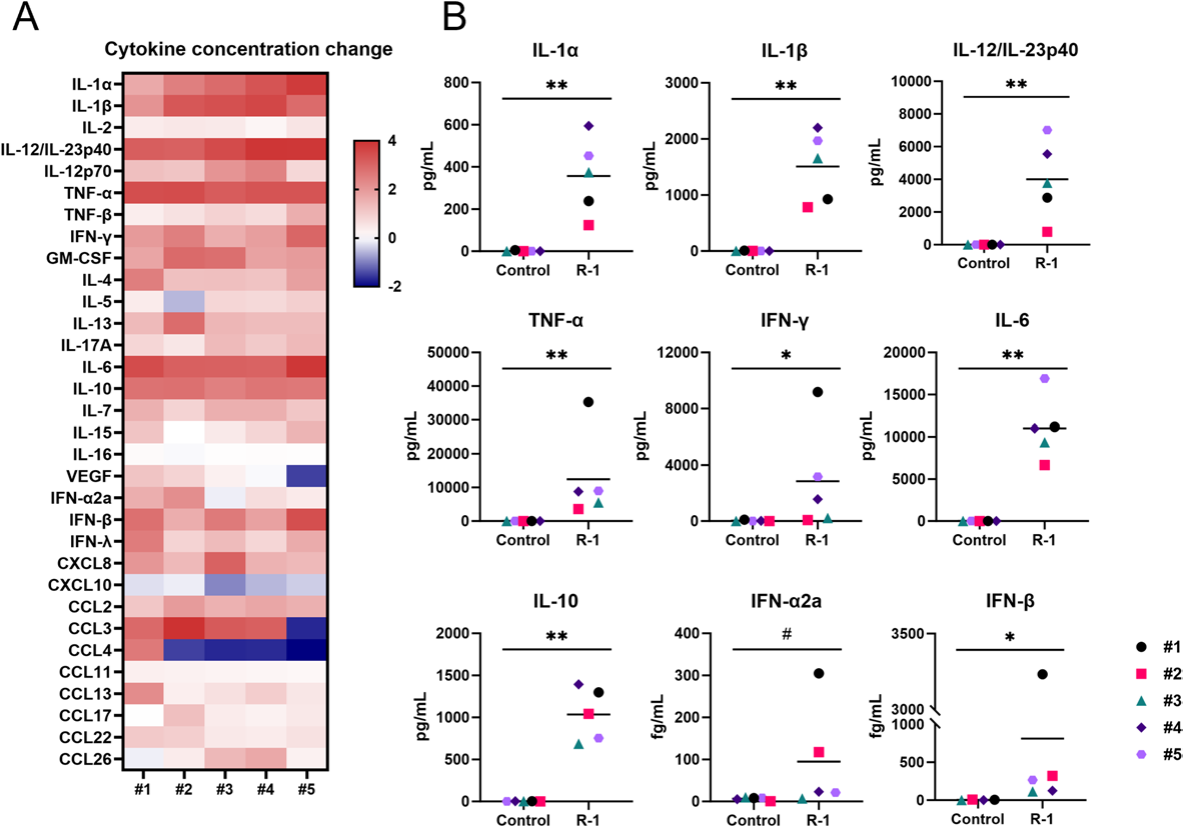
R-1 EPS induces proinflammatory cytokine production in peripheral blood mononuclear cells (PBMCs). Human PBMCs from five individual healthy donors were stimulated with 100 µg/mL of R-1 EPS for 24 h and cell-free supernatant (R-1 sup) were collected. Concentrations of 32 cytokines or chemokines in the PBMC supernatants were quantified. (**A**) Heatmap showing changes in the levels of 32 cytokines or chemokines after R-1 sup stimulation compared with the unstimulated PBMC supernatant (Control sup). Data are shown as log10 fold changes. (**B**) Representative individual cytokine and chemokine levels. Points in each plot represent the concentrations from individual donors (*n* = 1). Horizontal bars represent the mean concentrations from five donors. #: *P* < 0.1, *: *P* < 0.05, **: *P* < 0.01 by Mann–Whitney U test. Levels of other cytokines and chemokines are indicated in Supplementary Fig. S1.

PBMCs derived from five individual donors were used in the experiment. The gender, age, ethnicity, blood type, and body mass index (BMI) of donors are shown in Table 1. PBMCs were stimulated for 24 h with 100 µg/mL of R-1 EPS, and cell-free supernatants were collected (R-1 sup). In previous studies, stimulating PBMCs with R-1 EPS at concentrations of 100 µg/mL or higher significantly induced IFN-γ production, with the levels of IFN-γ production plateauing at concentrations above 100 µg/mL^21^. Therefore, in this study, a concentration of 100 µg/mL of R-1 EPS was used. Supernatants of unstimulated PBMCs were used as controls (Control sup). Multiplex analyses to quantify the expression of 32 cytokines and chemokines in the PBMC supernatant revealed that most were upregulated by R-1 EPS stimulation, whereas a few were suppressed (Fig. 1A). The extent of change caused by R-1 EPS stimulation was varied among donors, whereas the production of IL-1α, IL-1β, IL-12/IL-23p40, TNF-α, IL-6, IL-10 were significantly and strongly promoted in all donors, suggesting that proinflammatory response were induced in PBMCs by R-1 EPS stimulation (Fig. 1B, Supplementary Fig. S1). Despite very low secretion levels compared with other cytokines/chemokines (femto order), IFN-β, a well-known type I IFN that is critical for antiviral defense, was also significantly upregulated by R-1 EPS stimulation. IFN-α2a, another type I IFN, was upregulated in response to R-1 EPS stimulation; however, this increase was not statistically significant.

**Table 1:** Characteristics of the PBMC donors.

No.	Gender	Age	Ethnicity	Blood type	BMI
#1	Male	49	Caucasian	N/A	38
#2	Male	44	Caucasian	O+	20
#3	Male	23	Caucasian	O+	23
#4	Male	36	African American	O+	21
#5	Male	41	Caucasian	O-	29

## R-1 EPS-induced immune factors inhibit HCoV-229E replication

The anti-HCoV-229E effect of R-1 sup was examined in the human lung fibroblast cell line MRC5. MRC5 fibroblasts are a well-characterized, non-transformed human lung-derived cell line. Therefore, MRC5 cells may be suitable for evaluating innate immune responses, as they possess a functional interferon signaling pathway and are responsive to antiviral stimuli^22^. The use of primary cells related to coronavirus infection could also be considered; however, this was challenging in the present study owing to technical difficulties. In the viral growth inhibition assays, we confirmed that the treatment of MRC5 cells with R-1 sup from each donor, for 24 h, did not show any cytotoxicity in the WST-1 cell proliferation assay (Supplementary Fig. S2).

**Fig. 2:**
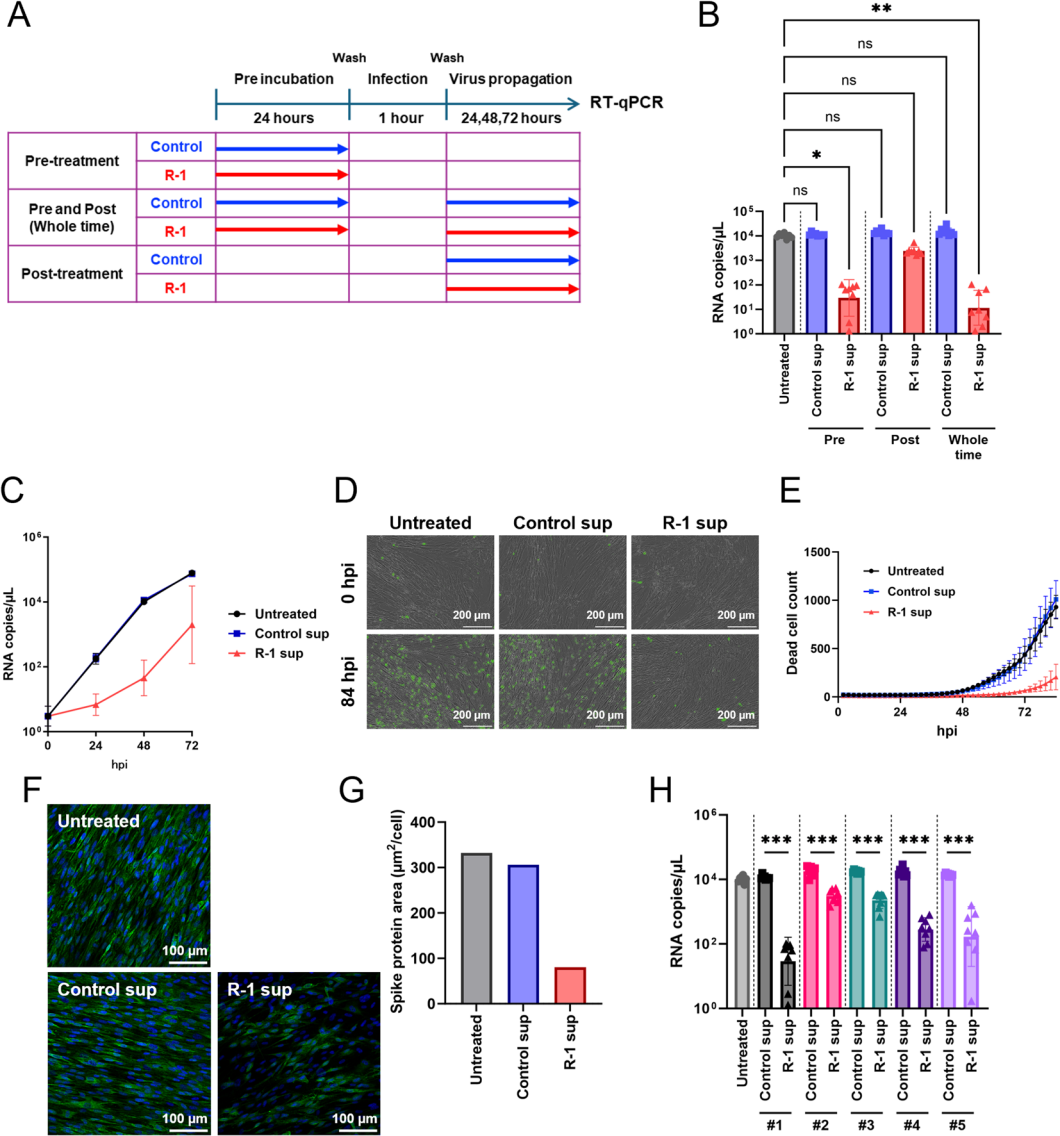
R-1 EPS-stimulated PBMC supernatant suppresses HCoV-229E replication. (**A**) Schematic diagram of PBMC supernatant treatment. (**B**) The antiviral effect of R-1 sup on HCoV-229E was evaluated using MRC5 as host cells. MRC5 cells were pre-, post-, or both pre- and post- (whole-time) treated by Control sup or R-1 sup (derived from donor #1) upon HCoV-229E infection. Viral genome RNA copy number in culture supernatant were titrated by RT-qPCR at 48 h post infection (hpi). Data are shown as mean ± SD (*n* = 8). *: *P* < 0.05, **: *P* < 0.01 by Kruskal-Wallis test. (**C**) The time course of HCoV-229E viral genome RNA replication in PBMC sup-pretreated cells. (**D**) Live-cell imaging in HCoV-229E infected MRC5 cells. Untreated, or Control sup or R-1 sup (derived from donor #1)-pretreated MRC5 cells were infected with HCoV-229E, and Incucyte^®^ Cytotox Reagents were applied to the cell culture medium and cell viability was monitored using Incucyte after infection. Merged images of phase contrast and Cytotox staining of dead cells (green) are shown. Scale bars indicate 200 μm. (**E**) Time course of the dead cell count in Live-cell imaging. Number of green-fluorescence positive dead cells were measured using the Incucyte software. Data are represented as mean ± SD (*n* = 8). (**F**) Immunostaining of HCoV-229E spike protein in virus infected cells (48 hpi), which were pretreated with Control or R-1 sup (derived from donor #4). Fluorescence of spike protein is shown in green and DAPI staining of cellular nuclear is shown in blue. Representative 20 × confocal images are shown. Scale bars indicate 100 μm. (**G**) Green-fluorescence positive spike protein area (µm^2^/cell) of HCoV-229E infected cells were measured. Bars represent average area of eight fields of view in each experimental condition. (**H**) Inhibitory effect of R-1 sup pretreatment on HCoV-229E replication were confirmed using PBMCs derived from five individual donors. Data are shown as mean ± SD (*n* = 8). ***: *P* < 0.001 by Mann–Whitney U test.

The antiviral effects of R-1 sup were evaluated for different stimulus timing (Fig. 2A). MRC5 cells were pre-, post-, or both pre- and post- (whole-time) treated with R-1 sup (derived from donor #1, arbitrarily selected from five donors) upon HCoV-229E infection, and the viral genome copy number in the culture medium was quantified at 48 h post-infection (hpi) by RT-qPCR. The results showed that the RNA copy number of HCoV-229E was significantly decreased under pre- and whole-time treatment conditions, but not under post treatment compared to that in untreated cells (Fig. 2B). Among these stimulus timing, there was little or no synergistic effect of both, pre- and post-treatment. The direct effects of R-1 EPS were also evaluated, revealing that neither pre-treatment nor post-treatment with R-1 EPS showed any inhibitory effect on viral replication (Supplementary Fig. S3). Orally administered R-1 EPS might exert its effects through immune cells rather than by activating cells in organs such as the lungs via circulation. Therefore, we consider the results of the model experiments using PBMCs to be more important than those obtained by directly exposing MRC5 cells to R-1 EPS. The kinetics of HCoV-229E replication over 72 hpi also showed that R-1 sup pre-treatment effectively suppressed viral replication between 0 and 48 hpi (Fig. 2C, Supplementary Fig. S4).

Moreover, because MRC5 cells clearly show cytopathic effect (CPE) after HCoV-229E infection^23^, we examined the effect of R-1 sup treatment on CPE. CPE cells were detected by fluorescence staining, indicating disruption of cell membrane integrity. The increase in CPE in untreated and Control sup-pretreated MRC5 cells was remarkable at 84 hpi, but slight in R-1 sup-pretreated cells (Fig. 2D, Supplementary video). CPE in cells was monitored every 2 h after HCoV-229E infection, using a live-cell imaging system, which showed that the appearance of dead cells was markedly suppressed in the R-1 sup-pretreated cells (Fig. 2E).

To further verify the suppressive effect of R-1 sup, immunostaining for the viral spike protein was performed in HCoV-229E infected MRC5 cells, before CPE was observed. Spike protein-expressing cells were decreased in R-1 sup-pretreated cells compared to untreated or Control sup-treated cells at 24 and 48 hpi (Fig. 2F, Supplementary Fig. S5). We quantified the spike protein expressing area by measuring the fluorescence-positive area using the image analysis software ImageJ Fiji^24^, which showed that the fluorescence-positive area was decreased in R-1 sup-pretreated cells (Fig. 2G, Supplementary Fig. S5). This confirmed the suppressive effect of R-1 sup on HCoV-229E replication.

Because pre-treatment with R-1 sup derived from donor #1 showed a notable suppressive effect on coronavirus replication, the inhibitory effects of R-1 sups derived from PBMCs from the other four donors were examined to consider the inter-donor variability in PBMC reactivity. As expected, the HCoV-229E viral genome copy number significantly decreased in all R-1 sup treated MRC5 cells (Fig. 2H), indicating that R-1 sup treatment of MRC5 inhibited HCoV-229E progeny production in the supernatant. The effect of R-1 sup treatment on CPE was also examined in the other four donors, and the results showed that the increase in CPE in cells was suppressed in R-1 sup-pretreated cells from all donors (Supplementary Fig. S6).

These results showed that pre-treatment of MRC5 cells with R-1 sup efficiently suppressed HCoV-229E replication in our in vitro experimental model.

## R-1 EPS-induced immune factors also inhibit SARS-CoV-2 replication

**Fig. 3:**
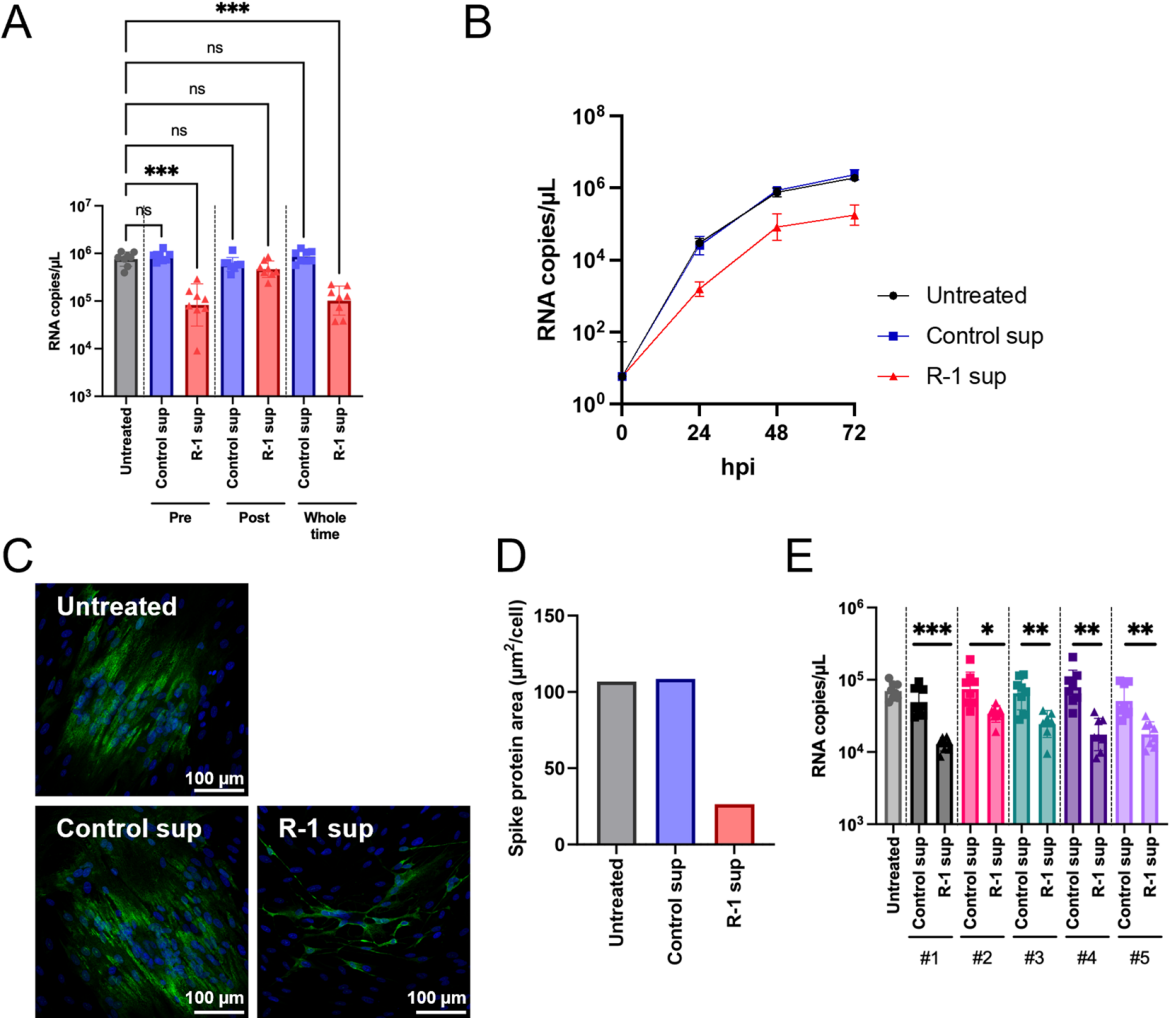
R-1 EPS-stimulated PBMC supernatant suppresses SARS-CoV-2 replication. The antiviral effect of R-1 sup on SARS-CoV-2 were evaluated using ACE2-expressing MRC5 (MRC5/hACE2) as host cells. (**A**) MRC5/hACE2 cells were pre-, post-, or both pre- and post- (whole-time) treated by Control sup or R-1 sup (derived from donor #1), upon SARS-CoV-2 infection. Viral genome RNA copy number in culture supernatant were titrated by RT-qPCR at 48 h post infection (hpi). Data are shown as mean ± SD (*n* = 8). ***: *P* < 0.001 by Kruskal-Wallis test. (**B**) Time course of SARS-CoV-2 viral genome RNA replication in PBMC sup-pretreated cells. (**C**) Immunostaining of SARS-CoV-2 spike protein in virus infected cells (24 hpi), which were pretreated with Control or R-1 sup (derived from donor #4). Fluorescence of spike protein is shown in green and DAPI staining of cellular nuclear is shown in blue. Representative 20 ×confocal images are shown. Scale bars indicate 100 μm. (**D**) Green-fluorescence positive spike protein area (µm^2^/cell) of SARS-CoV-2 infected cells were measured. Bars represent average area of eight fields of view in each experimental condition. (**E**) Inhibitory effect of R-1 sup pretreatment on SARS-CoV-2 replication were confirmed using PBMCs derived from five individual donors. Data are shown as mean ± SD (*n* = 8). *: *P* < 0.05, **: *P* < 0.01, ***: *P* < 0.001 by Mann–Whitney U test.

Following the finding that R-1 sup suppressed HCoV-229E replication, we further examined the suppressive effect of R-1 sup on SARS-CoV-2 infection. Although SARS-CoV-2 replication is limited in parental MRC5 cells due to low ACE2 expression, we transduced ACE2 to enable viral entry. This approach enabled consistent evaluation of antiviral activity in a defined cellular context. MRC5 cells stably expressing human ACE2 (MRC5/hACE2) were generated by transducing ACE2 gene with a lentiviral vector to MRC5 cells (Supplementary Fig. S7). MRC5/hACE2 cells were pre-, post-, or both pre- and post-(whole time) treated with R-1 sup (derived from donor #1) upon SARS-CoV-2 infection, and the viral genome copy number in the culture medium was quantified at 48 hpi using RT-qPCR. The RNA copy number of SARS-CoV-2 in the culture supernatant was significantly decreased in pre- and whole-time R-1 sup-treated MRC5/hACE2 cells, but not in post-treated cells (Fig. 3A). There was little or no synergistic effect between the pre- and post-treatment, as in the case of HCoV-229E. The kinetics of SARS-CoV-2 replication over 72 hpi showed that the inhibitory effect of R-1 sup pre-treatment was observed up to 24 hpi (Fig. 3B, Supplementary Fig. S8). Immunostaining of infected cells also revealed a remarkable reduction in the amount of spike protein in MRC5/hACE2 cells treated with the R-1 sup (Fig. 3C, D).

Subsequently, the inhibitory effects on SARS-CoV-2 were examined using supernatants derived from PBMCs of the five donors. The results indicated that R-1 sup from all the donors significantly suppressed the replication of SARS-CoV-2 (Fig. 3E). Taken together, these results show that the pre-treatment of R-1 sup suppressed the replication of SARS-CoV-2 as well as HCoV-229E. The finding that only pre-treatment, but not post-treatment, with R-1 sup exhibited inhibitory effects, suggests that R-1 EPS may have prophylactic potential against viral infection rather than therapeutic effects.

## R-1 sup enhances antiviral and inflammatory responses

To reveal the mechanisms underlying the antiviral effects of R-1 sup, transcriptomic analysis was performed. Since pre-treatment with R-1 sup suppressed coronaviral replication, it was suggested that the MRC5 cell antiviral response was enhanced prior to infection by R-1 sup pre-treatment. Thus, we conducted transcriptomic analysis of MRC5 cells pretreated with R-1 sup, but not infected.

**Fig. 4:**
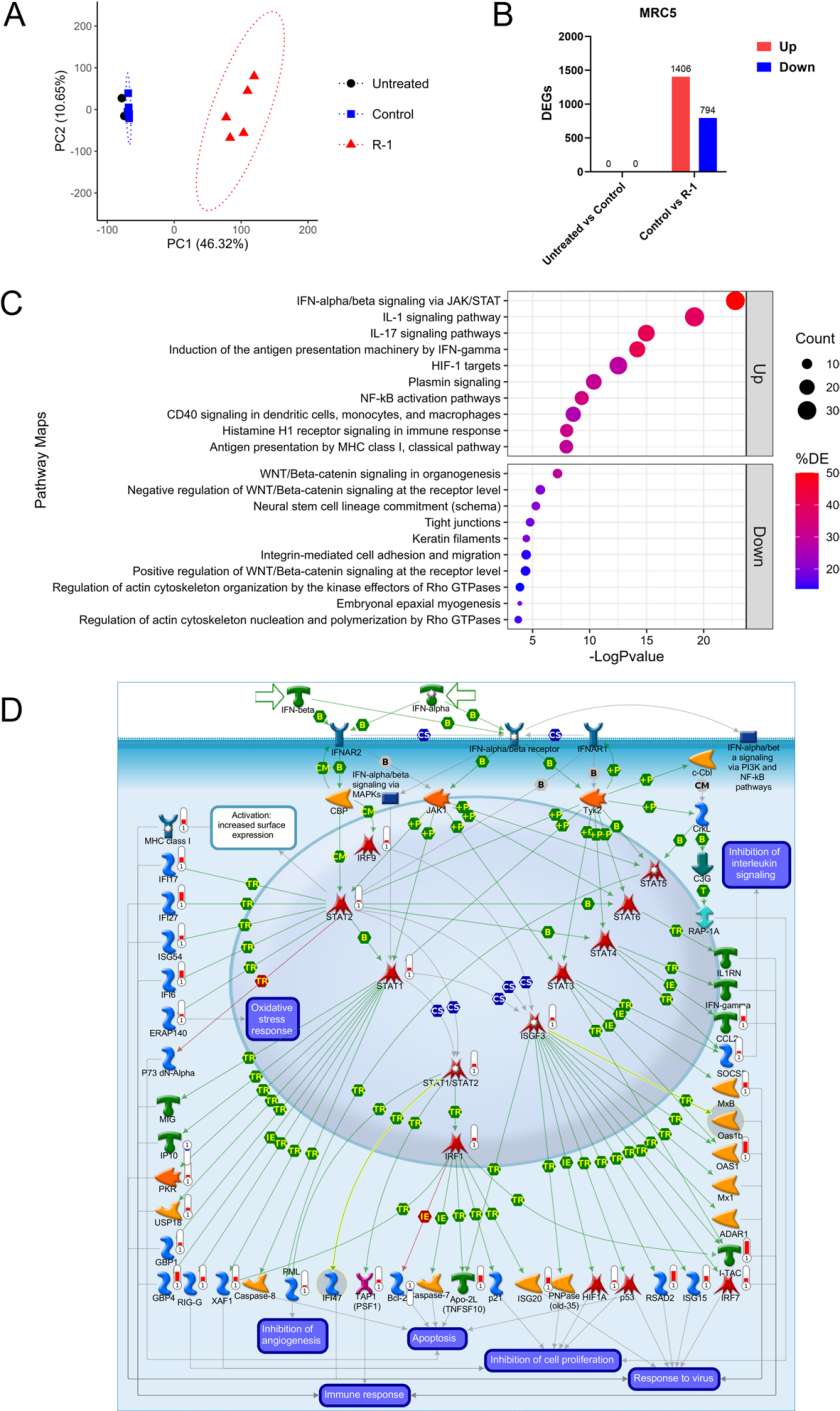
R-1 sup induces type I IFN and inflammatory responses in MRC5 cells. Cellular RNA was extracted from untreated, or Control sup or R-1 sup-treated MRC5 cells to perform RNA-Seq analysis. (**A**) Principal component analysis (PCA) was performed using normalized gene counts. Genes with low expression levels were excluded from the analyses. Each point represents an individual sample. Ellipses denote 95% confidence intervals assuming a multivariate normal distribution for each group. (**B**) The number of differentially expressed genes (DEGs, |log2 fold change| > 1, adjusted *P* value < 0.05) are indicated. (**C**) Top 10 enriched pathways of upregulated and downregulated DEGs by *P* value are shown (R-1 sup vs. Control sup). Pathway analysis was performed by MetaCore. Pathways are ranked by Log *P* values. Count indicates the number of DEGs in each pathway. Percent DE indicates the percentage of DEGs in the total object in each pathway. (**D**) Pathway map of “Interferon alpha/beta signaling via JAK/STAT pathway”. The meters next to the object indicate the DEGs in the dataset (R-1 sup vs. Control sup). Red and blue meters represent upregulated genes and downregulated genes, respectively. The level of the meter represents the intensity of the log2 fold change. Image generated using MetaCore. Detailed figure legends of pathway map are available in the MetaCore reference guide (https://portal.genego.com/legends/MetaCoreQuickReferenceGuide.pdf).

MRC5 cells were untreated or treated with Control or R-1 sup for 24 h and total RNA was extracted for RNA-Sequencing (RNA-Seq) analyses. PBMC supernatants derived from the five donors were used in this experiment. Read counts were normalized and genes with low expression levels were excluded from the dataset. Principal component analysis (PCA) showed that the cluster of R-1 sup-treated MRC5 cells was clearly distinguished from the clusters of untreated and Control sup-treated cells (Fig. 4A). Differential expression analyses showed that a total of 2,200 differentially expressed genes (DEGs) were detected, of which 1,406 genes were upregulated and 794 genes were downregulated when comparing R-1 sup-treated cells with Control sup-treated cells (Fig. 4B). In contrast, no DEGs were detected between the untreated cells and Control sup-treated cells. Based on these results, subsequent functional enrichment analyses were performed, focusing on the comparison between the Control sup- and R-1 sup- treated cells.

Gene ontology (GO) analysis showed upregulated DEGs were enriched in GO terms such as “defense response to virus,” “inflammatory response,” “immune response,” and “negative regulation of viral replication” (Supplementary Fig. S9). Pathway analysis was performed to explore differentially expressed molecular pathways, and the result showed “IFN-alpha/beta signaling via JAK/STAT” was the top ranked pathway by *P* value. “IL-1 signaling pathway,” “IL-17 signaling pathway,” and “Induction of the antigen presentation machinery by IFN-gamma” were the next ranked pathways, indicating that these pathways were upregulated in R-1 sup-treated cells (Fig. 4C, D, Supplementary Fig. S10). Among the DEGs enriched into the pathway “IFN-alpha/beta signaling via JAK/STAT”, signal transducer and activator of transcription (STAT) 1, STAT2 and interferon regulatory factor (IRF) 9, which are known to form interferon-stimulated gene factor 3 (ISGF3) complex essential for induction of interferon-stimulated genes (ISGs), and a number of ISGs were upregulated (Fig. 4D). These results suggest that R-1 sup induces an antiviral response, particularly type I IFN signaling, and an inflammatory response in MRC5 cells. Indeed, R-1 sups included IFNs.

Downregulated DEGs in MRC5 cells were enriched in GO terms such as “cell adhesion,” “intracellular signal transduction,” and “positive regulation of cell migration” (Supplementary Fig. S9). Pathway analysis showed that several enriched pathways were related to WNT/beta-catenin signaling, which regulates cell proliferation and differentiation, and Rho GTPases, which regulates cytoskeleton remodeling and cell migration (Fig. 4C). It is presumed that the high levels of inflammatory cytokines and chemokines in R-1 sup may have stressed the cultured cells, leading to the suppression of their cell functions. Several studies have reported that inflammatory cytokines inhibit the WNT/beta-catenin pathway and barrier function^25,26^. Although these pathways were downregulated in R-1 sup-treated cells, no obvious cytotoxicity was observed in R-1 sup-treated MRC5 cells (Supplementary Fig. S2). Thus, we concluded that the suppressive effect of R-1 sup on coronaviral replication was not due to its cytotoxicity. In contrast, it has been reported that infection with SARS-CoV-2 activates the WNT/beta-catenin pathway, and pharmacological inhibition of this pathway suppresses viral proliferation and enhances type I IFN production^27^. Therefore, the downregulation of the WNT/beta-catenin pathway may also be involved in the suppression of coronavirus replication. However, as the number of DEGs annotated to the WNT/beta-catenin pathway was limited, further validation is required to confirm its effect in this study.

Transcriptomic analyses were also performed on MRC5/hACE2 cells. PCA showed that a cluster of R-1 sup treated MRC5/hACE2 was clearly distinguishable from the clusters of untreated cells and Control sup-treated cells (Supplementary Fig. S11). A total of 1,854 DEGs (923 upregulated and 931 downregulated) were detected by comparing Control sup- versus R-1 sup-treated MRC5/hACE2, whereas 26 DEGs (11 upregulated and 15 downregulated) were detected by comparing untreated cells and Control sup-treated cells (Supplementary Fig. S11). These results indicated that treatment with R-1 sup induced changes in the transcriptome of MRC5/hACE2 cells, as in case of MRC5 cells.

The top enriched GO term by *P* value was “inflammatory response” and “response to virus” was ranked fifth (Supplementary Fig. S11). Pathway analysis showed “IL-1 signaling pathway” and “IL-17 signaling pathway,” which are pathways related to the signaling of inflammatory cytokines, ranked first and second, respectively. The pathway “IFN-alpha/beta signaling via JAK/STAT”, which was top ranked pathway by *P* value in MRC5, was ranked fourth (Supplementary Fig. S11). Several downregulated pathways were also related to WNT/beta-catenin signaling and Rho GTPases (Supplementary Fig. S11), similar to that of MRC5.

Thus, the transcriptome analysis revealed that R-1 sup treatment induced antiviral response in MRC5 and MRC5/hACE2 cells, leading cells into an antiviral state.

## R-1 sup induces an antiviral state in host cells

**Fig. 5:**
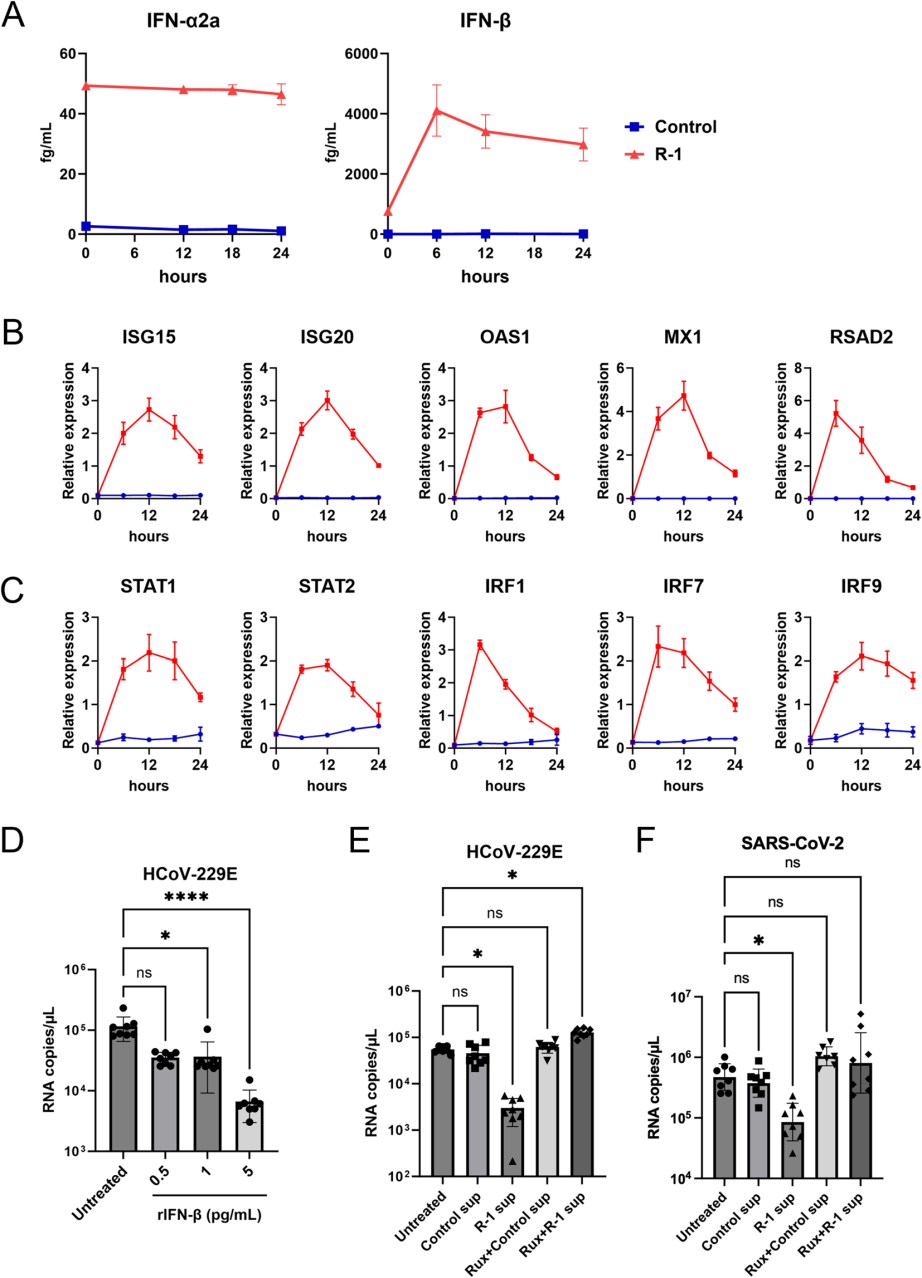
R-1 EPS-stimulated PBMC supernatant activates type I IFN signaling via JAK/STAT pathway in MRC5 cells. MRC5 cells were treated with Control sup or R-1 sup (derived from donor #1), and the concentrations of type I IFN in culture supernatant (**A**), gene expression levels of interferon stimulated genes (ISGs) (**B**), and signal transduction factors downstream of the IFN-alpha/beta receptor (**C**) were monitored every 6 h. The gene expression levels are shown relative to GAPDH expression. The red line and blue line indicate R-1 sup and Control sup-treated cells, respectively. (**D**) Inhibitory experiment for HCoV-229E replication using recombinant IFN-β. MRC5 cells were pretreated with 0.5, 1, and 5 pg/mL of recombinant human IFN-β (rIFN-β) for 24 h. Viral genome RNA copy number of HCoV-229E were quantified by qRT-PCR at 48 hpi. (E, F) Inhibitory experiment with IFN signaling using ruxolitinib (Rux), a JAK inhibitor, was performed. Rux was added to MRC5 or MRC5/hACE2 cells during the pre-treatment by R-1 sup. Viral genome RNA copy number of HCoV-229E (**E**) or SARS-CoV-2 (**F**) were quantified by qRT-PCR at 48 hpi. Data are shown as mean ± SD (*n* = 8). *: *P* < 0.05, ****: *P* < 0.0001 by Kruskal-Wallis test.

Transcriptome analysis indicated that antiviral gene expression, especially IFN signaling, was induced in R-1 sup-treated MRC5 cells. Indeed, IFN-α2a and IFN-β were produced from R-1 EPS-treated PBMCs in the medium (Fig. 1B, IFN-α2a and IFN-β). To further examine the activation of IFN pathway by monitoring IFN-α/β and ISGs production in MRC5 cells, cell culture supernatant was collected every 6 h after Control sup or R-1 sup treatment. The initial IFN-α2a and IFN-β concentrations (0 h) came from the cytokine concentration in the R-1 sup and Control sup, showing that the R-1 sup included higher levels of IFNs in PBMCs (Fig. 5A). The concentration of IFN-α2a in the medium remained constant for 24 h in R-1 sup-treated cells, indicating that the treatment with R-1 sup did not induce the production of IFN-α2a in MRC5 cells (Fig. 5A). In contrast, the production of IFN-β increased between 0 and 6 h, and although it slightly declined between 6 and 24 h, it remained at a higher concentration than at 0 h (Fig. 5A, IFN-β). This result suggests that the treatment with R-1 sup induced the production of IFN-β during 0 to 6 h in MRC5 cells. Accordingly, gene expression levels of ISGs (ISG15, ISG20, 2’-5’-oligoadenylate synthetase 1 [OAS1], MX dynamin like GTPase 1 [MX1], radical S-adenosyl methionine domain containing 2 [RSAD2]) and signal transduction factors downstream of the IFN-α/β receptor (STAT1, STAT2, IRF1, IRF7, IRF9) showed substantial increase within 6 h (Fig. 5B, C), whereas most gene expression levels were decreased from 6 to 12 h onwards till 24 h, concomitant with changes in IFN concentrations. Based on the results so far, we hypothesized that this amount of IFN-β induction by R-1 sup treatment may be sufficient for antiviral defense. Therefore, HCoV-229E infection was examined in MRC5 cells treated with each concentration of recombinant human IFN-β (rIFN-β). The maximal concentration of rIFN-β was set to 5 pg/mL, which is equivalent to the maximum concentration of IFN-β detected in the culture supernatant of MRC5 cells treated with R-1 sup (Fig. 5A). As a result, 5 pg/mL of rIFN-β significantly suppressed HCoV-229E replication despite its low concentration (Fig. 5D).

Since IFN-β was suggested to be an important factor for inhibiting viral replication by R-1 sup treatment, we attempted to inhibit IFN-α/β receptor (IFNAR)-JAK/STAT signaling pathway to see whether the effect of R-1 sup was impaired. Before viral infection, MRC5 cells were pretreated with 4 µM of ruxolitinib (Rux), a JAK inhibitor, and R-1 sup. Rux treatment completely abolished the suppressive effects of R-1 sup on HCoV-229E and SARS-CoV-2 replication (Fig. 5E, F). These results indicated that R-1 sup activated the type I IFN response via the JAK/STAT pathway in MRC5 cells.

Overall, our results clearly demonstrate that immune factors derived from R-1 EPS-stimulated PBMCs, induce an antiviral state in host cells by activating type I IFN signaling prior to infection and effectively suppress HCoV-229E and SARS-CoV-2 replication.

## Discussion

Public health has been repeatedly threatened by outbreaks of infectious diseases. With globalization, ongoing and future outbreaks of reemerging and newly emerging infectious diseases pose an undeniable risk. Under such circumstances, enhancing the innate human immune response is a crucial defense strategy for preventing infection. Utilization of functional foods that are sustainable, safe, and readily accessible is a valuable approach, and the use of functional foods, probiotics, and immunobiotics as therapeutic tools for COVID-19 has been suggested^28–30^. In this study, we investigated the antiviral effects of R-1 EPS and provide evidence for its potential use as a defense strategy against COVID-19.

We examined the antiviral effects of R-1 EPS on coronaviruses in an in vitro experimental model using PBMCs. In our model, R-1 EPS induced the production of cytokines and chemokines from PBMCs, and then, the anti-coronavirus effect of these factors was evaluated using a human lung cell line. Our experimental model presents a hypothesis regarding the mechanism of action of immunobiotic-mediated antiviral responses within the host, in which the immunobiotic initially interacts with immune cells and then enhances the antiviral activities of non-immune cells, such as fibroblasts and epithelial cells. In a mouse influenza model, oral administration of R-1 EPS results in an increase in antibody production in bronchoalveolar lavage fluid upon influenza virus infection. This suggests that the effects of orally administered R-1 EPS may act locally within tissues by acting immune cells and promoting cytokine production^18^. However, as the present study was conducted only in vitro, it remains unclear whether cytokines induced by R-1 EPS function in the lungs and exert antiviral effects in vivo. Further animal and clinical studies are required to establish the anti-coronaviral effects of R-1 EPS in vivo.

Our results showed that immune factors derived from R-1 EPS stimulated PBMCs activated type I IFN signaling in MRC5 cells, independent of infection. The levels of IFN-β in R-1 sup-treated MRC5 cells rapidly increased within 6 h, and gene expression level of ISGs also showed drastic but transient increase within 6 to 12 h, although the peak level of IFN-β in MRC5 supernatant was very low (5 pg/mL). The infection assay using recombinant protein showed IFN-β efficiently suppressed viral replication at the same concentration in vitro (Fig. 5D). Reports on the impact of IFN signaling on SARS-CoV-2 replication and COVID-19 pathology are increasing. Susceptibility of SARS-CoV-2 replication to type I IFN treatment appears to be consistent among some in vitro studies^31–34^. In contrast, for COVID-19 pathology, both protective and deteriorating effects of IFN signaling have been reported^35–39^. Some studies have shown that the timing of IFN treatment determines its effects on COVID-19 pathology. Rapid induction of type I IFN signaling in the early phase limits viral propagation and reduces mortality, but prolonged increases in type I IFN levels in the late phase of infection are associated with excess inflammation, delayed recovery, and increased mortality^37,40^. The results of our study showed that immune factors derived from R-1 EPS-stimulated PBMCs induced robust but transient type I IFN responses. Thus, this effect of R-1 EPS to enhance the antiviral response prior to infection potentially offers an efficient method for preventing SARS-CoV-2 infections in the early stages of infection. However, in our study, pretreatment suppressed viral replication, whereas post-treatment exerted no inhibitory effect. This finding suggests that R-1 EPS may have a prophylactic potential against viral infection but limited therapeutic efficacy after infection has occurred.

With the contribution of the broad-spectrum antiviral effects of type I IFN signaling, the antiviral effects of R-1 EPS on both HCoV-229E and SARS-CoV-2 were demonstrated in our study. It is presumed that R-1 EPS suppresses the replication of other SARS-CoV-2 variants. On the other hand, confocal microscopic analysis of SARS-CoV-2 infected cells at 24 hpi showed that fluorescence-positive cells in R-1 sup-treated MRC5 cells had a distinct cell shape compared to that of untreated or Control sup-treated cells, with a unique shrunken cytoplasm (Fig. 3C). This feature was not observed in HCoV-229E infected cells (Fig. 2F, Supplementary Fig. S5). Some studies have reported the protective effects of apoptosis and necroptosis in viral infection^41–43^; it is possible that cell death was selectively induced in SARS-CoV-2 infected cells by R-1 sup treatment, as a protective effect to prevent viral spread. However, we did not investigate the response of the host cells after viral infection in this study; therefore, our hypothesis remains speculative. Further analysis of the detailed impact of the suppressive effect of R-1 sup on viral multiplication and cellular response following viral replication may reveal the distinct suppression mechanisms of R-1 sup against HCoV-229E and SARS-CoV-2.

The molecular mechanisms of induction of IFN-β production in MRC5 cells by R-1 sup remains to be elucidated, but several studies have reported that some cytokines may be associated with induction of type I IFN. Taniguchi and Takaoka reviewed that a weak IFN-α/β signal transmitted independently of the viral infection stimulates amplified IFN production in response to viral infection^44^. However, the level of IFN-β detected in R-1 sup was considerably low at the femto-level, which lacks functional validation. Further investigation is necessary to validate whether this low concentration of IFN-β has the capacity to alter the immune response of MRC5 cells. With regard to other cytokines, MetaCore pathway of “IL-1 signaling pathway,” which was the pathway upregulated in R-1 sup treated MRC5 cells, indicated production of IFN-β may be induced downstream of IL-1R signaling via MAPK cascade and subsequent STAT1 and IRF1 activation (Supplementary Fig. S10). It is possible that R-1 sup directly enhanced the production of ISGs and type I IFN. It is well known that type I IFN signaling induces ISGF3 complex formation that directly binds to interferon-stimulated response element (ISRE), which is a transcriptional activator for ISGs, while type II IFN also leads γ-activated factor (GAF, homodimer of STAT1) formation to induce ISG expression^45^. Taken together, it is hypothesized that IFN-β, IL-1, IFN-γ and other cytokines or chemokines induced by R-1 EPS from PBMCs, contribute to IFN-β production and significant ISG expression, independent of viral infection. Future studies involving inhibitory assays with neutralizing antibodies or other agents to block the function of cytokines are required to identify the responsible cytokine or chemokine.

The production of cytokines and chemokines by PBMCs was drastically increased by R-1 EPS stimulation. The production of IL-1α, IL-1β, IL-12/IL-23p40, TNF-α, IL-6, and IL-10 were strongly promoted in all tested donors (Fig. 1). These cytokines are highly expressed in monocytes/macrophages^46^; hence, we inferred the effect of R-1 EPS on monocytes/macrophages. This is consistent with reports that R-1 EPS can activate the mouse macrophage cell line RAW264.7^47^. While the extent of increase varied between donors, IFN-γ level was also upregulated by R-1 EPS stimulation. The ability of R-1 EPS to promote IFN-γ production followed by activation of NK cell cytotoxicity are well studied in vitro, in vivo, and clinical studies^10,12,17,18,21,48^. The results of our study are consistent with those of previous studies. Moreover, transcriptome analysis showed that the MetaCore pathway “Induction of the antigen presentation machinery by IFN-gamma” were upregulated in R-1 sup-treated MRC5 cells (Supplementary Fig. S10). Taken together with the results of previous clinical studies, it may be concluded that the consumption of yogurt fermented with OLL1073R-1, augmented the serum antibody titer against the seasonal influenza vaccine^14^, suggesting the potential of R-1 EPS as a vaccine adjuvant. Further studies on immune cell activation, cellular defense responses, and the effect of R-1 EPS stimulation on acquired immunity are expected. Given that R-1 EPS is a metabolite of lactic acid bacteria and a mixture of components, its structural characteristics remain unclear. However, R-1 EPS includes an acidic polysaccharide (APS) and neutral polysaccharide (NPS)^11^. APS has a stronger ability than NPS to induce the production of IFN-γ and other proinflammatory cytokines^10,47^. Therefore, APS in R-1 EPS may interact with PBMCs to promote the production of proinflammatory cytokines or chemokines. Fractionation of R-1 EPS and further validation studies may help identify the active component mediating these effects. Prolonged induction of proinflammatory cytokines may pose potential risks by causing various symptoms. While mild cases often involve symptoms such as headache, diarrhea, fatigue, rash, arthralgias, and myalgias, severe cases can lead to organ dysfunction and other serious complications^49^. However, clinical trials involving the consumption of yogurt fermented with OLL1073R-1 have not reported such adverse events. We believe that the production of inflammatory cytokines induced by OLL1073R-1 or R-1 EPS in vivo is temporarily regulated. Nonetheless, comprehensive investigations into the regulation of inflammatory cytokine production will be necessary before the clinical use of R-1 EPS as an antiviral agent.

There are several limitations in our study. First, all five PBMC donors were male, aged 23–49; four of them had blood type O and one had an unknown blood type. Given the limited donor availability, we were unable to assess the effects of R-1 EPS in younger (< 23 years) or older (> 49 years) individuals, female individuals, or across different blood types. Second, we found that proinflammatory cytokines and chemokines produced by PBMCs pretreated with R-1 EPS contributed to the suppression of coronavirus replication. However, the specific cytokines responsible for the antiviral effect remain unknown. Future studies using neutralizing antibodies or other inhibitory agents against certain cytokines will be necessary to determine the key factor involved. Third, this study was conducted in vitro, and the antiviral effects of R-1 EPS or OLL1073R-1 in vivo have not been verified. Further studies are required to determine if these agents can exert antiviral effects against coronaviruses in animal models or humans.

In conclusion, the results presented in this study provide evidence that the immunomodulatory EPS derived from *Lactobacillus delbrueckii* ssp. *bulgaricus* OLL1073R-1 enhances the antiviral immune response in host cells and suppresses the replication of SARS-CoV-2 and HCoV-229E. Furthermore, we also propose that the induction of type I IFN followed by ISG expression is involved in this mechanism. The effect of R-1 EPS, which induces an antiviral immune response, potentially offers an efficient method for preventing early stage SARS-CoV-2 infection. Further mechanistic studies and clinical trials are necessary to establish the beneficial role of R-1 EPS, in the development of defense strategies against SARS-CoV-2 infection.

## Methods

### Cells

MRC5 fatal lung normal diploid fibroblasts (CCL-171, ATCC; Manassas, VA, USA) and human peripheral blood mononuclear cells (PBMC, purchased from Precision for medicine, MD, USA) were maintained in Eagle’s minimal essential medium (EMEM) with L-glutamine, phenol red, sodium pyruvate, non-essential amino acids and 1,500 mg/L sodium bicarbonate (Fujifilm Wako Pure Chemical, Osaka, Japan) supplemented with 10% heat-inactivated fetal bovine serum (FBS, Biowest, Nuaillé, France), 100 U/mL penicillin and 100 µg/mL streptomycin (Gibco, Waltham, MA, USA). Cells were cultivated at 37 ℃ under 5% CO_2_-humidified atmosphere.

MRC5/hACE2 cells were established by the lentiviral transduction of pLVSIN-Hyg-hACE2 into MRC5 cells, which was purchased from ATCC. Cells were maintained in MEM (Nacalai Tesque, Kyoto, Japan) containing 10% heat-inactivated FBS, 100 U/mL penicillin/streptomycin (Nacalai Tesque), and 100 µg/mL hygromycin B (Nacalai Tesque) at 37 ℃, supplied with 5% CO_2_.

### Virus

Human coronavirus 229E (HCoV-229E, VR-740, ATCC) was propagated in MRC5 cells. HCoV-229E were infected into 80–90% confluent MRC5 cells and incubated in EMEM containing 2% FBS without antibiotics, at 35 °C with 5% CO_2_, for 5 days. After incubation, the culture supernatants were collected. Cellular debris were removed by centrifugation, and supernatants containing the HCoV-229E virus were stored at −80 °C. Infectious viral titers were determined using the 50% tissue culture infectious dose (TCID_50_). SARS-CoV-2, KUH003 (accession number LC630936)^50^ was isolated from nasal swabs of patients with COVID-19, in Tokyo.

### Purification of exopolysaccharides

The R-1 EPS was prepared according to a previously described method^17^. Briefly, skim milk fermented with *Lactobacillus delbrueckii* ssp. *bulgaricus* OLL1073R-1 was mixed with trichloroacetic acid to remove proteins, and ethanol precipitation was performed. Crude R-1 EPS was dialyzed (molecular weight cut-off: 6,000–8,000 Da) and incubated with RNase, DNase, and proteinase K. After ethanol precipitation and dialysis, the R-1 EPS was obtained by freeze-drying. For use in the experiments, freeze-dried R-1 EPS was suspended in the appropriate medium for each experiment and dissolved completely by incubating at 4 °C, overnight.

### PBMC supernatant preparation

Cryopreserved PBMCs were thawed, suspended in EMEM supplemented with 10% FBS, 100 U/mL penicillin, and 100 µg/mL streptomycin, and incubated at 37 °C with 5% CO_2_. After overnight incubation, cells were collected by centrifugation, and seeded in 48-well tissue culture treated plates at a concentration of 5 × 10^5^ cells/500 µL/well. Cells were stimulated with 100 µg/mL of R-1 EPS for the appropriate time, in each experiment (6-, 12-, 18-, and 24-h for IFN-α2a, β quantification, and gene expression analyses in MRC5 cells, and 24 h for other assays). Unstimulated PBMC supernatant was used as a control. After stimulation, the plates were centrifuged and the supernatants were collected. The collected supernatants were centrifuged again to completely remove the cellular debris. To calculate the change in cytokine concentration, the cytokine concentrations of the R-1 EPS-stimulated PBMC supernatant were divided by the concentrations of the control supernatant, and the data were shown as log10 fold changes. The lowest detectable concentration was used to calculate the change in the undetectable cytokine concentration.

### Multiplex analyses of cytokines and chemokines

Cytokine and chemokine levels in PBMCs or MRC5 culture supernatants were determined with the U-PLEX Custom Biomarker (hu) Assays kit (for IL-29/IFN-λ1), S-PLEX Human kit (for IFN-α2a and IFN-β), and V-PLEX Human Cytokine 30-Plex Kit (for other tested cytokines and chemokines), using the Meso QuickPlex SQ120 instrument (Meso Scale Discovery; MSD, Rockville, MD, USA), according to the manufacturer’s protocol.

### HCoV-229E infection

MRC5 cells were seeded in 96-well plates and cultured until 80–90% confluent. Cells were washed with EMEM containing 2% FBS before use in the assay. For pre- or whole treatment, the cells were treated with 6-fold diluted PBMC supernatants for 24 h at 37 ℃. For untreated control and post treatment, cells were incubated with EMEM with 2% FBS for 24 h at 37 ℃. After preincubation, the cells were washed with EMEM with 2% FBS, and infected with HCoV-229E at multiplicity of infection (MOI) = 0.05 and incubated for an hour at 35 ℃. The cells were washed to remove unbound viruses and EMEM with 2% FBS was added with (post- and whole time) or without (pre-) PBMC supernatants and incubated for 24-, 48-, and 72-h at 35 ℃. The supernatants were used to determine the number of viral RNA copies by RT-qPCR (SARS-CoV-2 Detection Kit -N2 set, TOYOBO, Japan), according to the manufacturer’s protocol; however, the primers and probe were replaced with the specific primers and probe for HCoV-229E (forward primer: 5′-TGCTGCGGCTCTTAAATCTT-3′, reverse primer: 5′-GGCTTTTGCATTTCATGCTT-3′, probe: FAM-5′-TGCCAAGAGTCTTGCTCGTTCTCAG-3′-MGB Eclipse). For inhibition of IFN signaling, the cells were treated with 6-fold diluted PBMC supernatants and 4 µM ruxolitinib for 1 h, at 37 ℃, before viral infection.

### SARS-CoV-2 infection

The MRC5/hACE2 cells were seeded in 96-well plates and cultured until they reached 100% confluence. For pre- or whole treatment, the cells were treated with 6-fold diluted PBMC supernatants for 24 h at 37 ℃. For untreated control and post treatment, cells were incubated with 2% MEM for 24 h at 37 ℃. After preincubation, the cells were washed with 2% MEM and infected with SARS-CoV-2 at MOI = 0.05 and incubated for an hour at 37 ℃. The cells were washed to remove unbound viruses and 2% MEM was added with (post- and whole time) or without (pre-) PBMC supernatants and incubated for 24-, 48-, and 72-h at 37 ℃. The supernatants were used to determine the number of viral RNA copies using RT-qPCR (SARS-CoV-2 Detection Kit -N2 set, TOYOBO, Japan), according to the manufacturer’s protocol. For inhibition of IFN signaling, the cells were treated with 6-fold diluted PBMC supernatants and 4 µM ruxolitinib for 1 h at 37 ℃, before viral infection.

### HCoV-229E inhibition assay using recombinant IFN-β

MRC5 cells were seeded in 96-well plates and cultured until 80–90% confluent. Cells were washed with EMEM containing 2% FBS before use in the assay. Recombinant human IFN-β (092–06061, rIFN-β, Fujifilm Wako Pure Chemical), at concentrations 0.5, 1, and 5 pg/mL, was added to culture medium and incubated for 24 h at 37 ℃. After incubation, the cells were washed with EMEM with 2% FBS and infected with HCoV-229E at MOI = 0.05 and incubated for an hour at 35 ℃. The cells were washed to remove unbound viruses and EMEM with 2% FBS was added and incubated for 48 h at 35 ℃. Supernatants were used to determine the number of viral RNA copies by RT-qPCR, as described for HCoV-229E infection.

### Immunofluorescence staining of coronavirus-infected cells

Cells infected at 24 and 48 hpi with HCoV-229E and 24 hpi with SARS-CoV-2 were fixed in cold methanol for 20 min. After drying and rinsing with PBS, two drops of Image-iT FX Signal Enhancer (Thermo Fisher Scientific, Waltham, MA, USA) were added and incubated for 30 min at room temperature (RT). The cells were blocked with 2% normal goat serum for 20 min at RT and labeled with anti-SARS-CoV-2 Spike (40591-T62; Sino Biological, Beijing, China) or anti-HCoV-229E Spike (MAB10938; R&D Systems, Minneapolis, MN, USA) antibodies for 2 h, followed by incubation for an hour, with fluorescein-conjugated goat anti-IgG (ab150113 for HCoV-229E, ab150077 for SARS-CoV-2; Abcam, Cambridge, UK). Cellular nuclear were stained with ProLong™ Gold Antifade Mountant with 4’,6-diamidino-2-phenylindole (DAPI; Thermo Fisher Scientific). The cells were visualized using a confocal laser scanning microscope (LSM880; Zeiss, Oberkochen, Germany). The spike fluorescence-positive area was measured and DAPI fluorescence-positive cellular nuclei were counted using ImageJ Fiji^24^. Fluorescent images of eight fields for each experimental condition were obtained, and the average area was calculated.

### Live-cell imaging using incucyte live-cell analysis system

The viral infection assay was performed as described below. For dead cell staining, Incucyte Cytotox Reagents (Sartorius, Göttingen, Germany) were added to the culture medium after infection, and the cells were monitored using the Incucyte S3 Live-Cell Analysis system (Sartorius) every 2 h from 0 hpi until 84 hpi. To measure the number of dead cells, fluorescent images of nine fields per well were taken, and the number of green fluorescence-positive dead cells was measured using Incucyte analysis software. The average of nine fields was used as the number of dead cells per well.

### RNA-Seq

MRC5 cells were either untreated or treated with control PBMC supernatant or R-1 EPS-stimulated PBMC supernatant for 24 h. PBMCs derived from five donors were used for the experiment. Total RNA was extracted from MRC5 cells using a RNeasy Mini Kit (QIAGEN, Hilden, Germany). Libraries were generated from purified RNA samples (> 7 RNA integrity numbers [RIN]) using an Illumina Stranded mRNA Prep Ligation kit (Illumina, San Diego, CA, USA) and sequenced on an Illumina Next-Seq 2000 sequencer (Illumina) according to the manufacturer’s protocol.

### RNA-Seq data processing

Read count data for each sample were obtained using the Illumina DRAGEN RNA version 4.0.4 and DRAGEN Differential Expression version 4.0.3 applications (Illumina). The read count data were transformed into counts per million (CPM) for normalization, and PCA was performed. CPM greater than 0.5 in at least one library were included in the analysis, and other genes were excluded because of low expression levels. PCA was performed using R (v4.3.1)^51^, and visualization was performed using the R package ggplot2^52^.

Differential expression analyses were performed using iDEP.96 (http://bioinformatics.sdstate.edu/idep/)^53^ by the DEseq2 method. Genes with low expression were excluded from the dataset using the same criteria as that used for the PCA. Genes with expression levels changed more than 2-fold (log2 fold change > 1 or log2 fold change < − 1), and an adjusted *P* value < 0.05 between the two groups compared were defined as DEGs. GO analyses were performed using DAVID (https://david.ncifcrf.gov/home.jsp)^54,55^, and pathway analyses were performed using MetaCore (MetaCore, a Cortellis solution, https://portal.genego.com/ (accessed on 15 September 2023)). Visualization was performed using the R packages ggplot2^52^.

### Gene expression analysis in MRC5 cells

Total RNA was extracted using an RNeasy Mini Kit (QIAGEN), and complementary DNA (cDNA) was prepared using PrimeScriptTM RT Master Mix (Takara Bio, Ohtsu, Japan) according to the manufacturer’s protocol. RT-qPCR was performed using TB Green^®^ Premix Ex Taq™ II (Takara Bio) with Quantstudio^®^ 3 system (Thermo Fisher Scientific). Glyceraldehyde 3-phosphate dehydrogenase (GAPDH) was used as the reference gene. Primers used are listed in Table 2.

**Table 2:** Specific primers and probe oligonucleotides for RT-qPCR.

Target gene	Forward/Reverse	Sequence (5′−3′)
GAPDH	Forward	CACCATCTTCCAGGAGCGAG
	Reverse	TCACGCCACAGTTTCCCGGA
ISG15	Forward	GCGAACTCATCTTTGCCAGT
	Reverse	AGCATCTTCACCGTCAGGTC
ISG20	Forward	CACCCCTCAGCACATGGT
	Reverse	TGGAAGTCGTGCTTCAGGT
OAS1	Forward	CTGGTGCTGAAACTGAAGAAAC
	Reverse	TACCTCTGAAGCATCCGAAATC
MX1	Forward	AGAGCCTCATCCGCCTAGTCAA
	Reverse	GCTCCCAAGCATAGACCGTCA
RSAD2	Forward	TGGTGAGGTTCTGCAAAGTAG
	Reverse	GTCACAGGAGATAGCGAGAATG
STAT1	Forward	CAGAGCTCGTTTGTGGTGGA
	Reverse	CATGGAAGTCAGGTTCGCCT
STAT2	Forward	GGAATCAGGCATGTGTCCCTT
	Reverse	TTCACCTCTCACCCCAATGGA
IRF1	Forward	CCTTAAGAACCCGGCAACCT
	Reverse	TGCATCTCTAGCCAGGGTCT
IRF7	Forward	TGGTCCTGGTGAAGCTGGAA
	Reverse	GATGTCGTCATAGAGGCTGTTGG
IRF9	Forward	TAGCCTATGCATGGCACTCC
	Reverse	AAAGGCCTGCTCCATCTTCA

### Statistical analysis

All statistical analyses were performed on GraphPad Prism version 10.0 for Windows (GraphPad Software). Statistical significance was set at *P* < 0.05.

## Supplementary Information

Below is the link to the electronic supplementary material.

Supplementary Material 1

Supplementary Material 2

## Data Availability

Raw RNA-Seq data were deposited in the National Center for Biotechnology Information (NCBI) with the primary accession code GSE295798. The other data presented in this study are available upon request from the corresponding author.
