# Supplementary material for: Immune factors produced by PBMCs upon stimulation with lactobacillus delbrueckii ssp. bulgaricus OLL1073R-1-derived exopolysaccharides inhibit HCoV-229E and SARS-CoV-2 replication
Source: Sci Rep. 2025 Aug 27;15:31621. doi: 10.1038/s41598-025-17308-3 (PMC12391341; doi:10.1038/s41598-025-17308-3)
Supplement: Supplementary file 2 — Supplementary Material 2 [file 41598_2025_17308_MOESM2_ESM.pdf]

Supplementary Information

Immune factors produced by PBMCs upon stimulation with *Lactobacillus delbrueckii* ssp. *bulgaricus* OLL1073R-1-derived exopolysaccharides inhibit HCoV-229E and SARS-CoV-2 replication

Shuyi Tang[1, *, †], Reiko Takai-Todaka[2, †], Shoko Ishii[1, **], Hiroki Kono[1, **], Reiko Watanabe[1, **], Miho Ogawa[1, **], Hiroshi Kano[1, **], Kazuhiko Katayama[2], Toshihiro Sashihara[1, **], Kenichi Hojo[1, *], Kei Haga[2, ‡]

[1] Food Microbiology and Function Research Laboratories, R&D division, Meiji Co., Ltd., 1-29-1 Nanakuni, Hachioji, Tokyo 192-0919, Japan

[2] Laboratory of Viral Infection Control, Ōmura Satoshi Memorial Institute, Graduate School of Infection Control Sciences, Kitasato University, 5-9-1, Shirokane, Minato-ku, Tokyo 108-8641, Japan.

[*] Present affiliation: Health Science Research Unit, R&D Division, Meiji Co., Ltd., 1-29-1 Nanakuni, Hachioji, Tokyo 192-0919, Japan

[**] Present affiliation: Wellness Science Labs, Meiji Holdings Co., Ltd., 1-29-1 Nanakuni, Hachioji, Tokyo 192-0919, Japan

[†] Equally contributed to this work.

[‡] Corresponding author: Kei Haga , Email: khaga@lisci.kitasato-u.ac.jp

Supplementary Methods

Cell proliferation assay

Cell viability of PBMC supernatant-treated MRC5 cells were measured using Premix WST-1 Cell Proliferation Assay System (Takara Bio, Shiga, Japan) according to the manufacturer's protocol. Briefly, MRC5 cells were either untreated, or treated with Control or R-1 sup for 24 h. After PBMC sup treatment, the medium was replaced with a medium containing 10% premix WST-1 reagent, and the absorbances at 450 nm and 690 nm were measured at 0, 1, 2, and 4 h after WST-1 reagent was added to MRC5 cells.

Evaluation of the direct antiviral effects of R-1 EPS

MRC5 cells were seeded in 96-well plates and cultured until 80-90% confluent. Cells were washed with EMEM containing 2% FBS before use in the assay. For pre-treatment, MRC5 cells were treated with 6-fold diluted 100 µg/mL of R-1 EPS (final concentration at 16.7 µg/mL) for 24 h at 37 °C. For untreated control and post treatment, cells were incubated with EMEM with 2% FBS for 24 h at 37 °C. After preincubation, the cells were washed with EMEM with 2% FBS, and infected with HCoV-229E at multiplicity of infection (MOI)=0.05 and incubated for an hour at 35 °C. The cells were washed to remove unbound viruses and EMEM with 2% FBS was added with (post-treatment) or without (untreated control and pre-treatment) R-1 EPS and incubated for 48 h at 35 °C. The supernatants were used to determine the number of viral RNA copies by RT-qPCR (SARS-CoV-2 Detection Kit -N2 set, TOYOBO, Japan), according to the manufacturer's protocol; however, the primers and probe were replaced with the specific primers and probe for HCoV-229E (forward primer: 5'-TGCTGCGGCTCTTAAATCTT-3', reverse primer: 5'-GGCTTTTGCATTTCATGCTT-3', probe: FAM-5'-TGCCAAGAGTCTTGCTCGTTCTCAG-3'-MGB Eclipse).

Western blotting

Whole-cell lysates were prepared by adding Laemmli's SDS-PAGE sample buffer after washing the cells with PBS (-) solution. Lysates were separated on a 5–20% polyacrylamide gel (ATTO, Tokyo, Japan) and transferred onto a polyvinylidene fluoride (PVDF) membrane (Bio-Rad Laboratories, Hercules, CA, USA) using Trans Blot Turbo (Bio-Rad Laboratories). An anti-hACE2 antibody (Atlas Antibodies, Stockholm, Sweden) was used as the primary antibody. HRP-conjugated anti-rabbit IgG (Cell Signaling Technology, Danvers, MA, USA) was used as the secondary antibody. Anti-actin hFAB rhodamine (Bio-Rad Laboratories) was used to detect actin expression. ChemiDoc Touch (Bio-Rad Laboratories) was used to detect the proteins visualized using Chemi-Lumi One L Solution A and Chemi-Lumi One L Solution B (Nacalai Tesque, Kyoto, Japan).

Supplementary figure S1

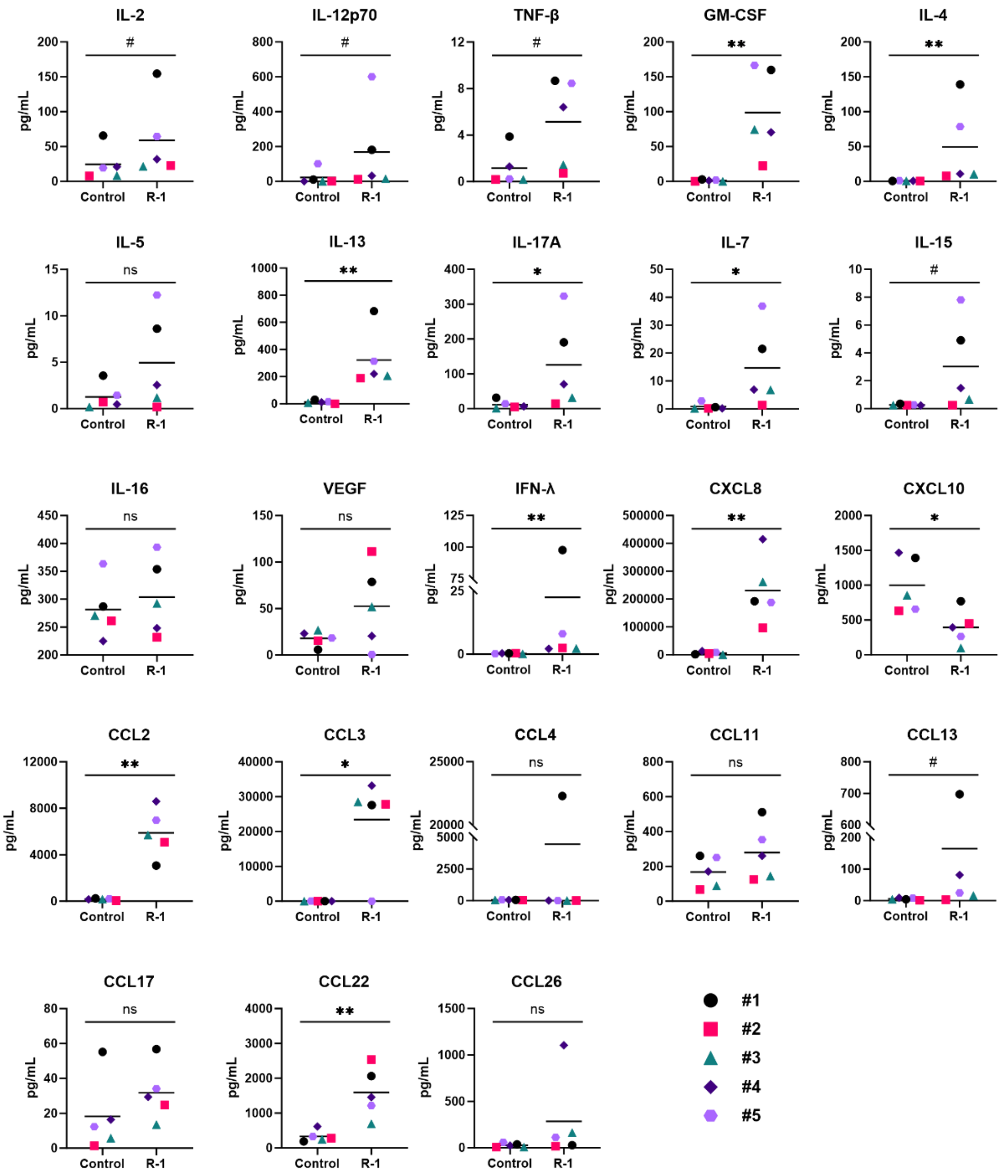

Supplementary Figure S1. Concentrations of cytokines and chemokines in R-1 EPS-stimulated PBMC supernatant. Points in each plot represent the concentrations in PBMC supernatants from the individual donors (n = 5). The bars represent the mean concentration of the five donors. #: $P < 0.1$, *: $P < 0.05$, **: $P < 0.01$ by Mann–Whitney U test. Data not shown in Fig. 1 in the original paper, are presented here.

Supplementary figure S2

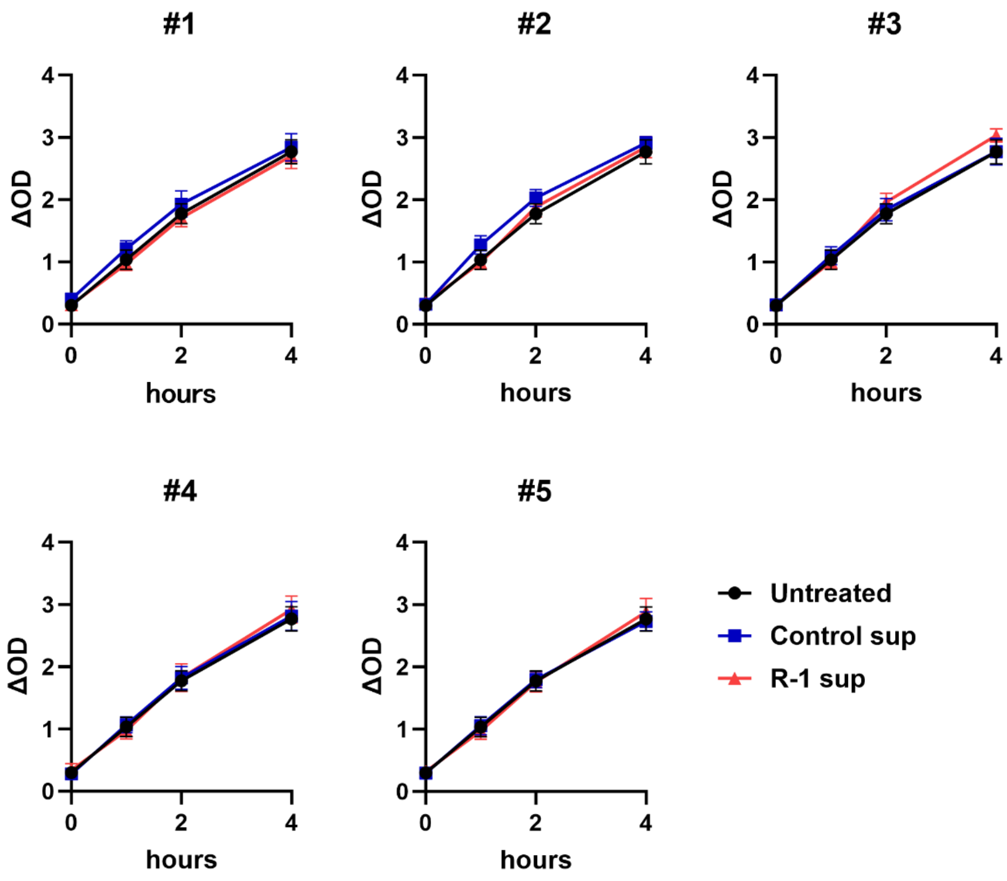

Supplementary Figure S2. WST-1 cell proliferation assay of R-1 sup-treated MRC5 cells. Cell viability of untreated MRC5 cells or MRC5 cells after 24 h of treatment with Control or R-1 sup was measured using the WST-1 assay. Absorbance at 450 nm and 690 nm was measured at 0, 1, 2, and 4 h after the addition of WST-1 reagent to MRC5 cells. Data are shown as mean ± SD (n = 8).

Supplementary figure S3

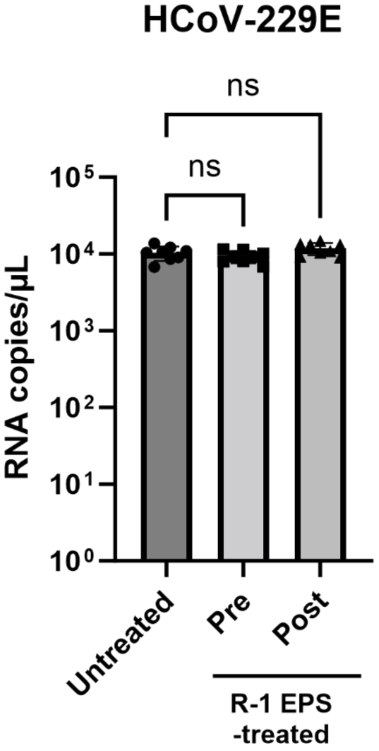

Supplementary Figure S3. R-1 EPS-treatment shows no directory effect on MRC5 cells.

The directory antiviral effect of R-1 EPS on HCoV-229E was evaluated using MRC5. MRC5 cells were pre- or post- treated by R-1 EPS upon HCoV-229E infection. Viral genome RNA copy number in culture supernatant were titrated by RT-qPCR at 48 hours post infection (hpi). Data are shown as mean ± SD (n = 8). No significant difference was detected by Kruskal-Wallis test.

Supplementary figure S4

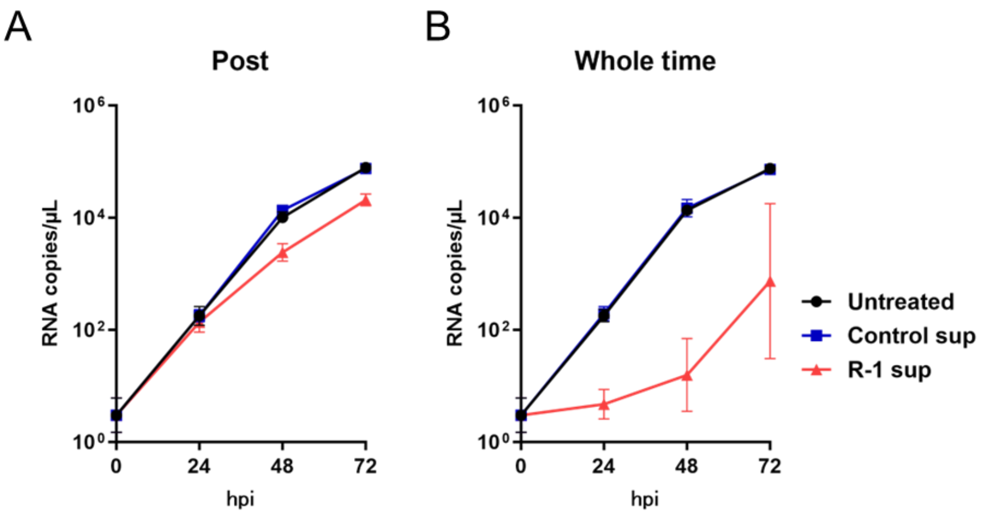

Supplementary Figure S4. Kinetics of HCoV-229E replication in post- or whole-time-treated MRC5 cells.

Kinetics of HCoV-229E replication in untreated, Control sup, or R-1 sup post- (A) or whole-time- (B) treated MRC5 cells. Viral infections were conducted at an MOI of 0.05, and the viral genome RNA copy number in the culture supernatant was titrated using qRT-PCR at 0, 24, 48, and 72 hpi. Data are shown as mean ± SD (n = 8).

Supplementary figure S5

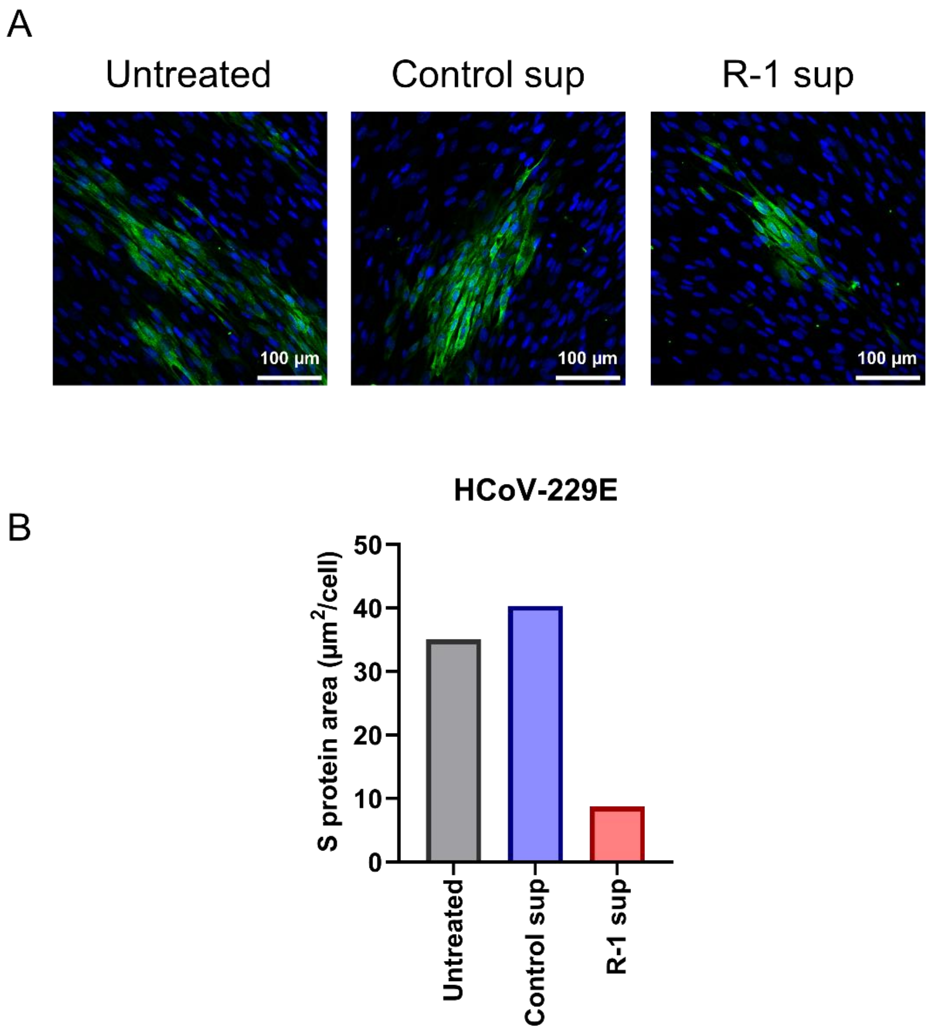

Supplementary Figure S5. Immunostaining of HCoV-229E-infected MRC5 cells.

(A) Immunostaining of HCoV-229E (24 hpi) spike protein in virus infected cells, which are pretreated with Control or R-1 sup (derived from donor #4). Fluorescence of the spike protein is shown in green and DAPI staining of cellular nuclear is shown in blue. Representative 20 × confocal images are shown. Scale bars indicate 100 µm. (B) Green-fluorescence positive spike protein area (µm^2/cell) in HCoV-229E infected cells were measured using ImageJ. Bars represent average area of eight fields of view, in each experimental condition.

Supplementary figure S6

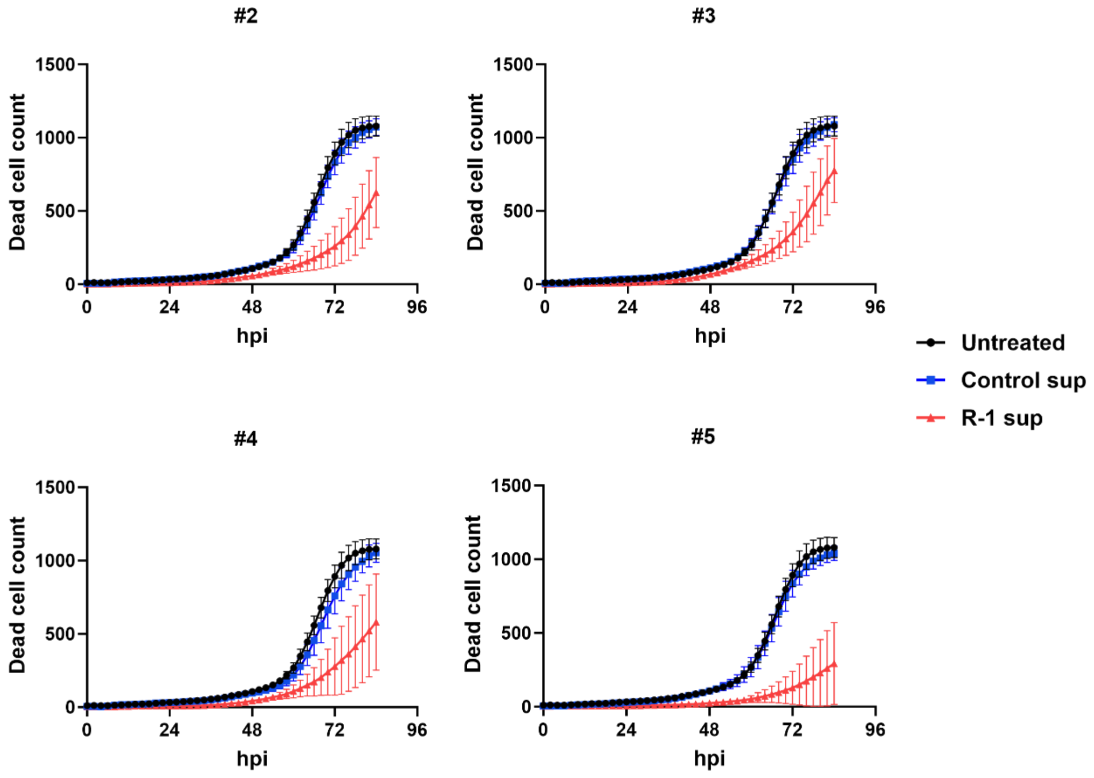

Supplementary Figure S6. Dead cell count of HCoV-229E infected cells.

Untreated and Control sup- or R-1 sup (derived from donors #2–#5)-pretreated MRC5 cells were infected with HcoV-229E. After infection, Incucyte® Cytotox Reagents were applied to the cell culture medium and cell viability was monitored using Incucyte. The number of green fluorescent-positive dead cells was measured using the Incucyte software. Data are represented as mean ± SD (n = 8).

Supplementary figure S7

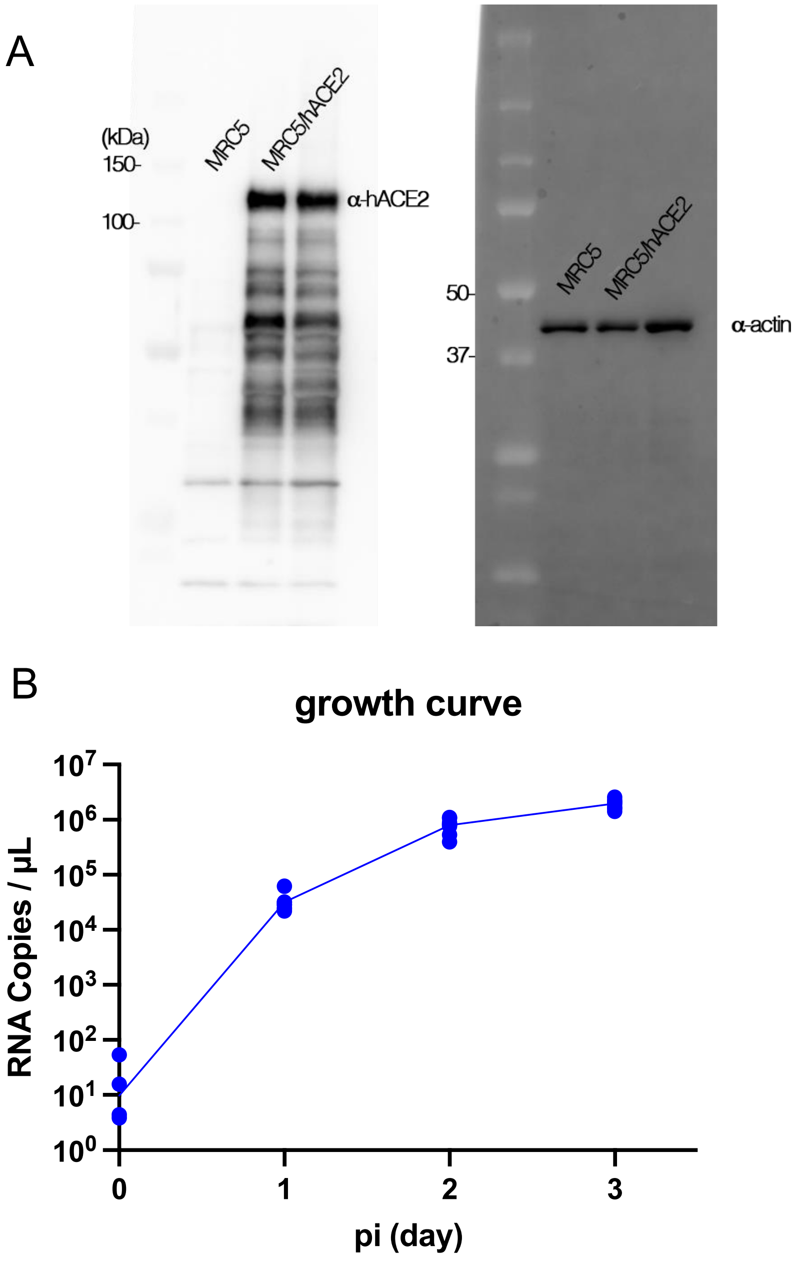

Supplementary Figure S7. Production of MRC5/hACE2 cells.

(A) Western blotting analysis of human ACE2 expression on MRC5/hACE2 cells. (B) Growth curve of SARS-CoV-2 in MRC5/hACE2 cells.

Supplementary figure S8

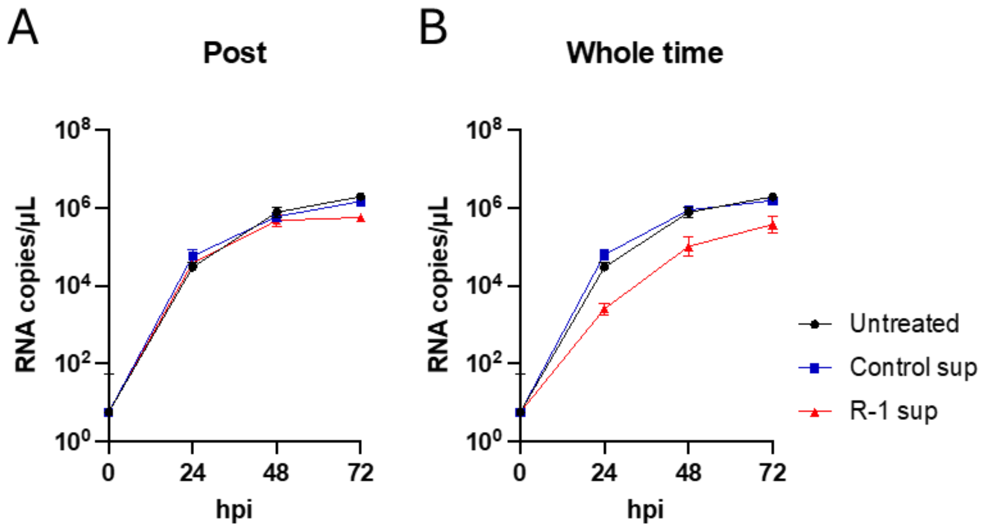

Supplementary Figure S8. Kinetics of SARS-CoV-2 replication in post- or whole-time-treated MRC5 cells.

Kinetics of SARS-CoV-2 replication in untreated, Control sup, or R-1 sup post- (A) or whole-time (B) treated MRC5 cells Viral infections were conducted at an MOI of 0.05, and the viral genome RNA copy number in the culture supernatant was titrated using qRT-PCR at 0, 24, 48, and 72 hpi. Data are shown as mean ± SD (n = 8).

Supplementary figure S9

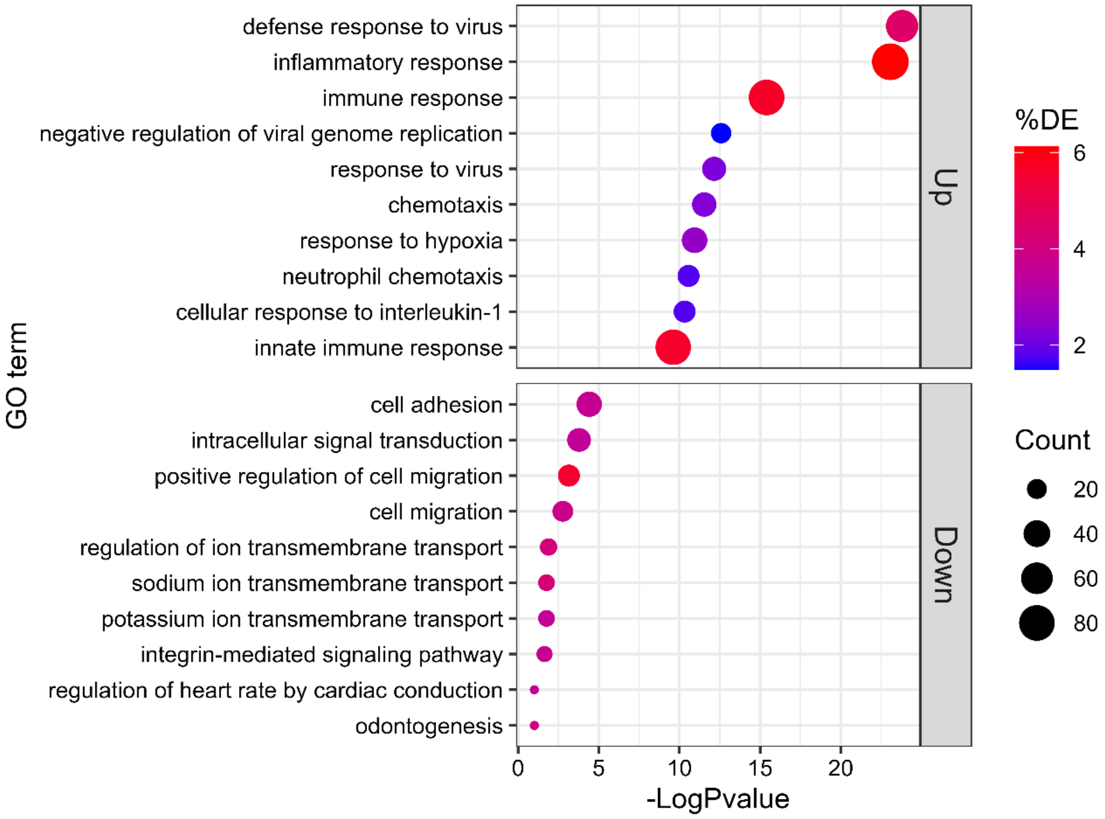

Supplementary Figure S9. Top 10 enriched GO terms in R-1 sup-treated MRC5 cells.

The top 10 enriched GO terms of upregulated and downregulated differentially expressed genes (DEGs) by *P* value are shown (R-1 sup vs. Control sup). GO analysis was performed using the DAVID software. GO terms were ranked by Log *P* values. Counts indicate the number of DEGs in each pathway. The percentage DE indicates the percentage of DEGs annotated to each GO term among the total DEGs.

Supplementary figure S10

A

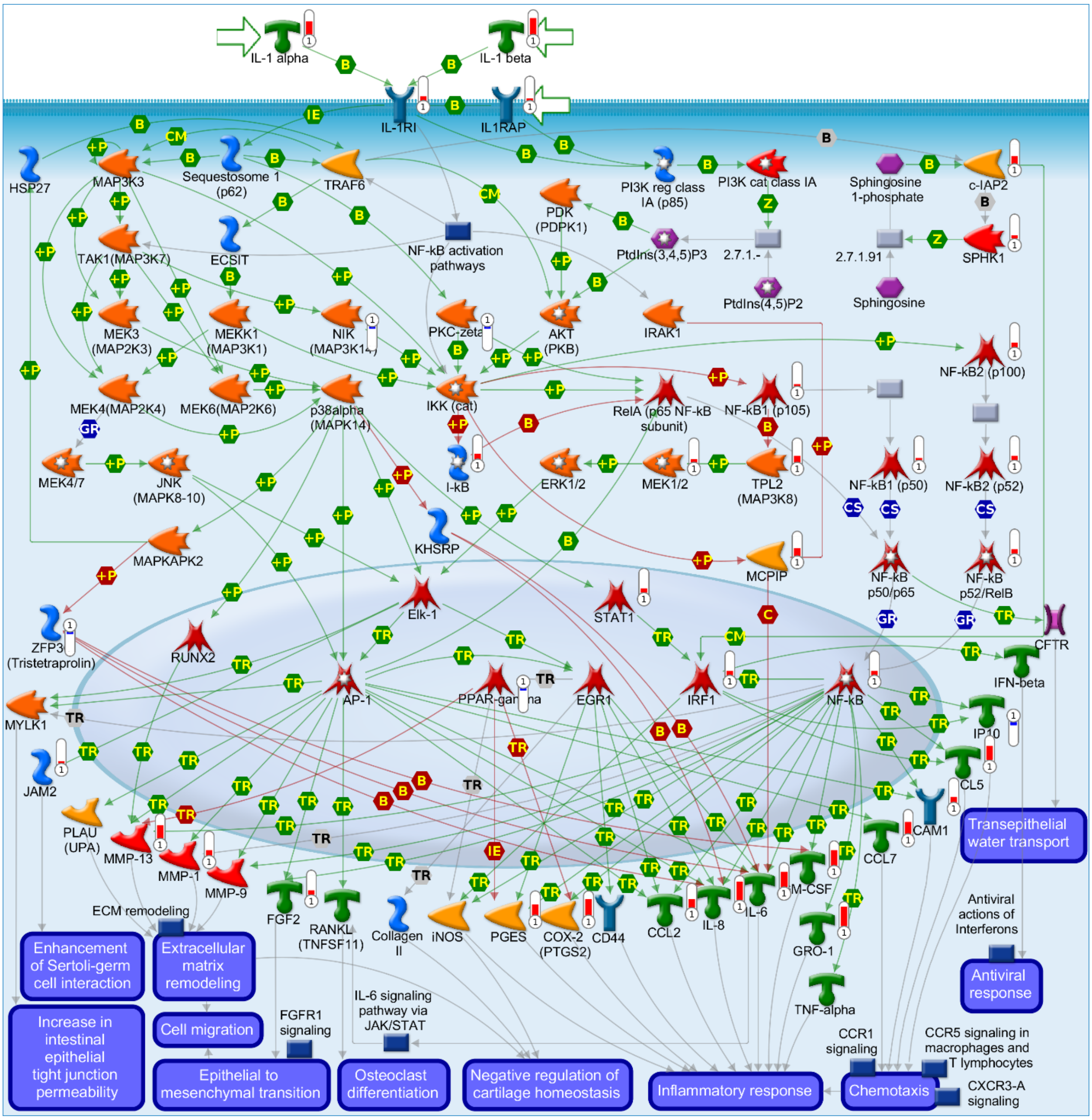

B

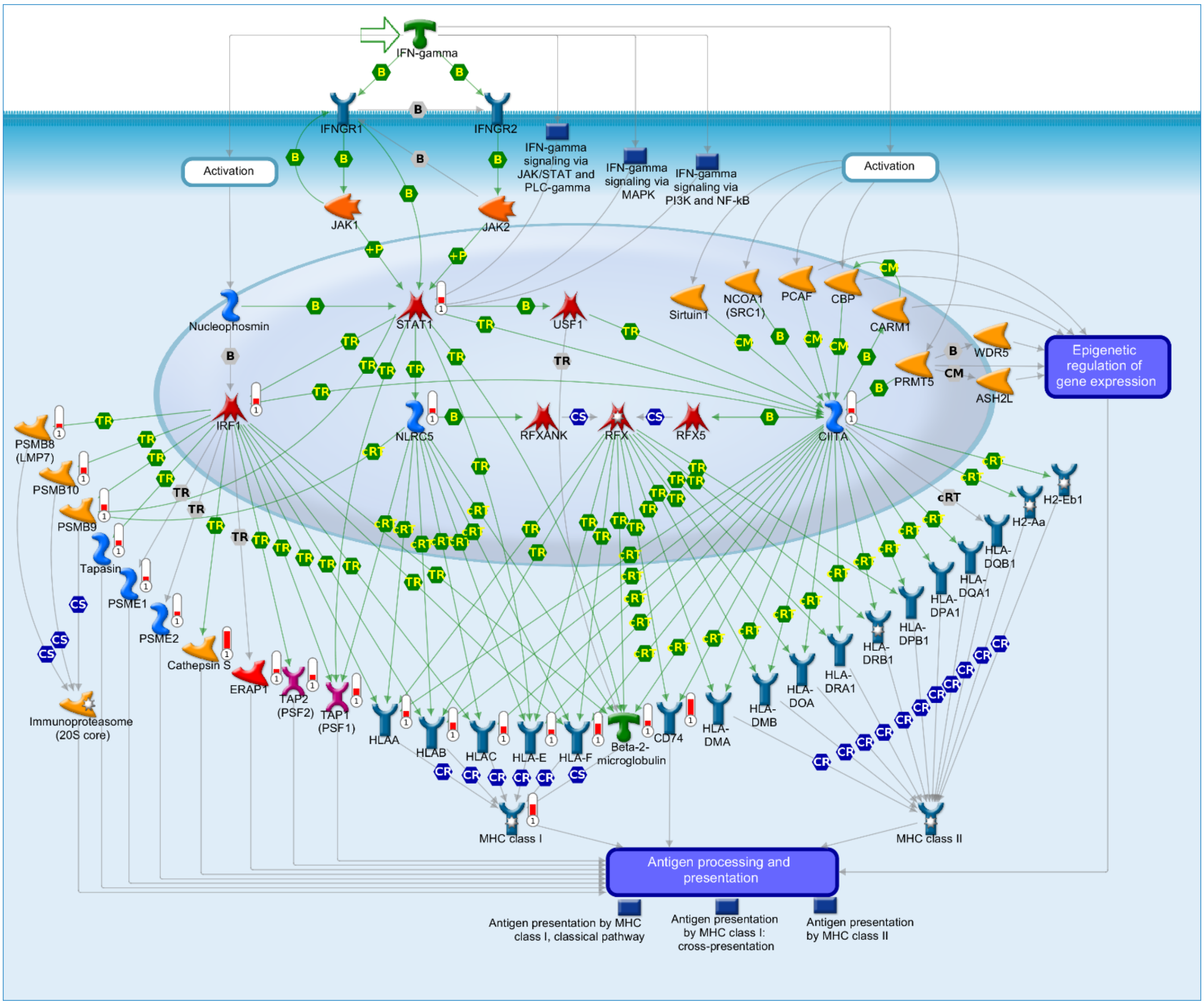

Supplementary Figure S10. MetaCore pathway map of upregulated pathways in R-1 sup-treated MRC5 cells.

(A) "IL-1 signaling pathway", (B) "Induction of the antigen presentation machinery by IFN-gamma". The meters next to the object indicate the DEGs in the dataset (R-1 sup vs. Control sup). Red meter and blue meter represent upregulated genes and downregulated genes, respectively. The level of the meter represents the intensity of the log2 fold change. Image generated using MetaCore. Detail figure legends of pathway map are available in the MetaCore reference guide (https://portal.genego.com/legends/MetaCoreQuickReferenceGuide.pdf).

Supplementary figure S11

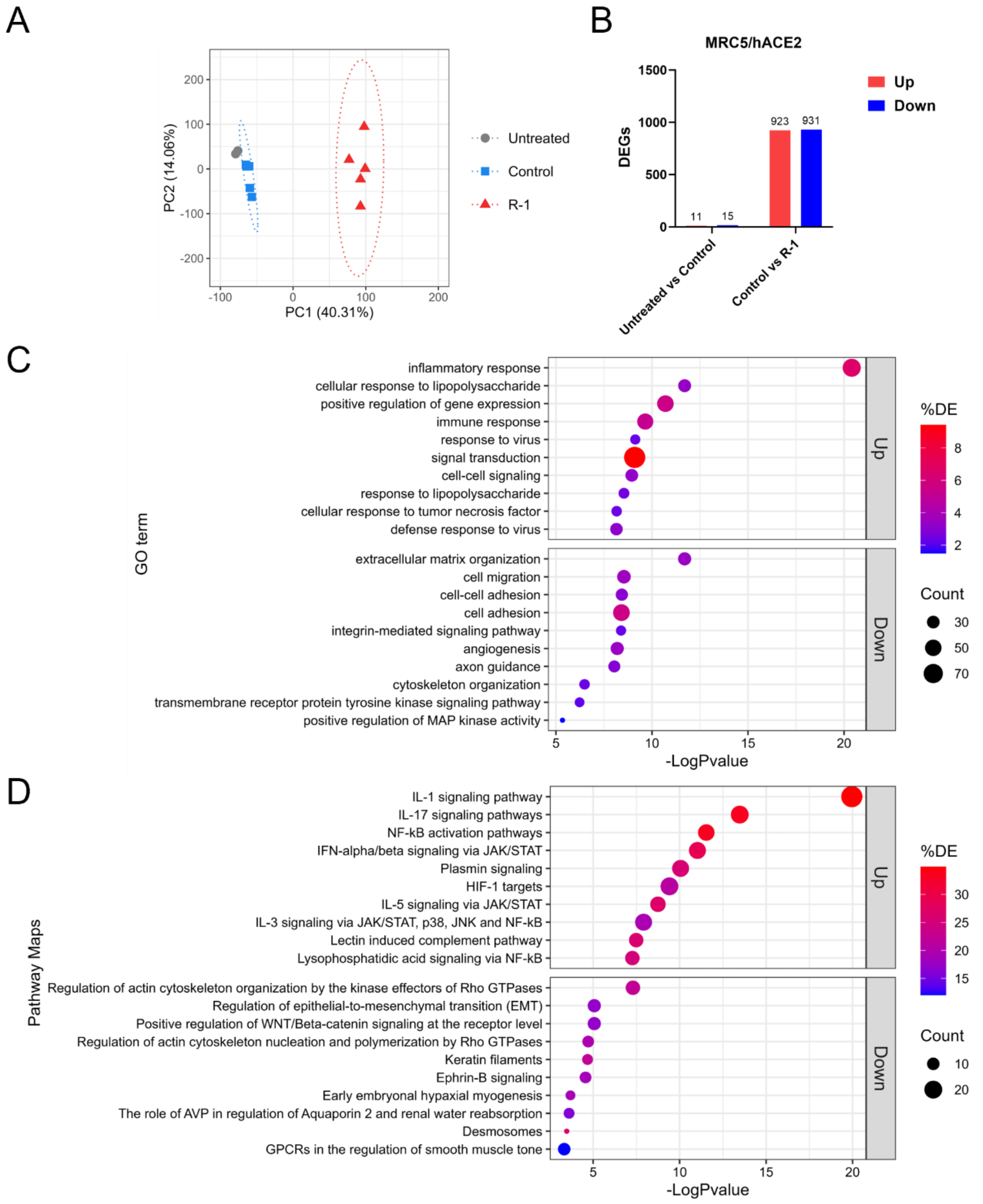

Supplementary Figure S11. Transcriptomic analyses of R-1 sup-treated MRC5/hACE2 cells. Cellular RNA was extracted from untreated, Control sup or R-1 sup-treated MRC5/hACE2 cells for RNA-Seq analysis. (A) Principal component analysis (PCA) was performed using normalized gene counts. Genes with low expression levels were excluded from the analyses. Each point represents an individual sample. Ellipses denote 95% confidence intervals assuming a multivariate normal distribution for each group. (B) The number of differentially expressed genes

(DEGs, |log2 fold change| > 1, adjusted *P* value < 0.05) are indicated. (C) Top 10 enriched GO terms of upregulated and downregulated DEGs by *P* value are shown (R-1 sup vs. Control sup). GO analysis was performed using the DAVID software. GO terms are ranked by Log *P* values. Count indicates the number of DEGs in each GO term. Percent DE indicates the percentage of DEGs annotated to each GO term among the total DEGs. (D) Top 10 enriched pathways of upregulated and downregulated DEGs by *P* value are shown (R-1 sup vs. Control sup). Pathway analysis was performed by MetaCore. The pathways are ranked by Log *P* values. Count indicates the number of DEGs in each pathway. Percent DE indicates the percentage of DEGs in the total objects in each pathway.

Supplementary video

Live-cell imaging in HCoV-229E infected MRC5 cells. Control sup or R-1 sup (derived from donor #1)-pretreated MRC5 cells were infected with HCoV-229E, and IncucyteR Cytotox Reagents were applied to the cell culture medium and cell viability was monitored using Incucyte. after infection. The video shows time-lapse images captured every two hours. Merged images of phase contrast and Cytotox staining of dead cells (green) are shown. Scale bar indicates 100 μm.
